# Visium spatial transcriptomics reveals intratumor heterogeneity and profiles of Gleason score progression in prostate cancer

**DOI:** 10.1016/j.isci.2023.108429

**Published:** 2023-11-10

**Authors:** Yongjun Quan, Hong Zhang, Mingdong Wang, Hao Ping

**Affiliations:** 1 Department of Urology, Beijing Tongren Hospital, Capital Medical University, Beijing 100176, China; 2 Department of Pathology, Beijing Tongren Hospital, Capital Medical University, Beijing 100176, China

**Keywords:** Medicine, Cancer, Transcriptomics

## Abstract

Prostate cancer (PCa) frequently presents as a multifocal disease within a single gland. Herein, the transcriptome-wide profiles of glandular epithelial (GE) cells of four PCa tissues with various Gleason scores (GSs) are analyzed with Visium spatial transcriptomics (ST). The genetic classifications across PCa section sites generally matched the spatial patterns of histological structures with different GSs. Average inferred copy number variation (inferCNV) values gradually increased during GS development. Developing trajectories during GS upgrading were assessed, and differentially expressed genes (DEGs) during GS progression were analyzed which exhibited heterogeneity among individual patients with PCa. Several crucial genes, such as NANS, PABPC1L, PILRB, PPFIA2, and SESN3, were associated with GS upgrading. Enrichment analysis showed that biological functions, such as cadherin binding, Golgi vesicle transport, protein folding, and cell adhesion molecules were related to GS progression. In conclusion, this study provides insight into ST-based transcriptome-wide expression patterns during GS progression.

## Highlights

• Accurately evaluated the transcriptome-wide levels of glandular epithelial (GE) cells
• Found crucial genes related to Gleason score (GS) progression of prostate cancer (PCa)
• Revealed individual heterogeneity in the transcription levels of different PCa

## Graphical abstract

**Figure:**
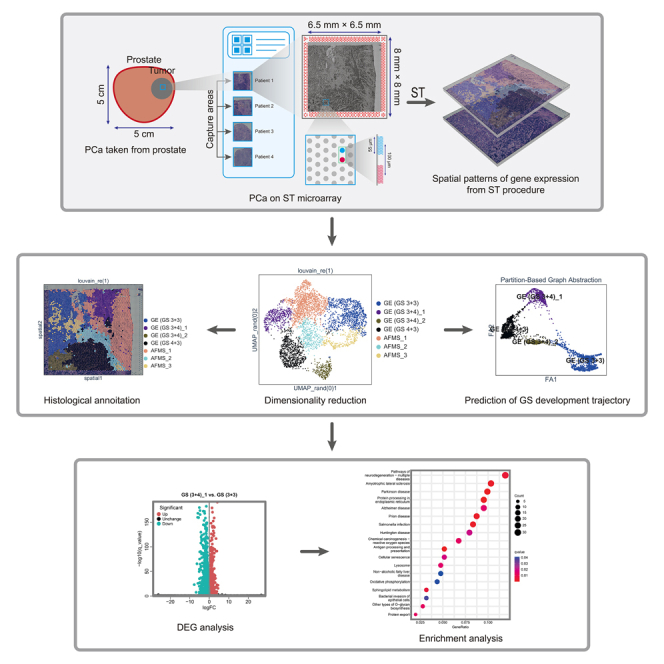

## Introduction

Prostate cancer (PCa) is the most common malignancy among men, with an estimated 288,300 new cases (29%, ranked first among all cancers) and 34,700 associated deaths (11%, ranked second among all cancers) in the United States in 2023.^1^

PCa frequently presents as multifocal disease within a single gland,^2^^,^^3^ and there is no envelope separating it from normal tissue. The clonal evolution of this intratumor heterogeneity is widely studied, and more recent studies tend to indicate that multifocal disease is derived from a monoclonal origin.^4^^,^^5^^,^^6^^,^^7^^,^^8^ Investigating the relationship of genetic patterns within multiple PCa lesions helps us to track the origins and drivers of PCa evolution.

In multifocal PCa, it is crucial to evaluate the grade of malignancy of each lesion. Despite the development of radiological technology and biomarkers, the histologic differentiation grade is still the strongest predictor of PCa malignancy, and the Gleason grading system for PCa is the most reliable evaluative criterion for pathological severity.^9^^,^^10^^,^^11^ The Gleason system is an architectural grading system (ranging from 1 to 5, for well to poorly differentiated) that enables a standardized risk assessment of patients with PCa with extraprostatic extension, biochemical recurrence, and overall survival.^12^^,^^13^^,^^14^^,^^15^^,^^16^^,^^17^ The Gleason score (GS) is obtained from the primary and secondary patterns of the Gleason grading system (range, 2 to 10), where GS ≥ 7 is associated with a greater risk of aggressiveness and a worse prognosis than GS < 7.^18^^,^^19^

The search for the origin of PCa is still ongoing, and luminal or basal cells are believed to be the origin cells of PCa.^20^^,^^21^^,^^22^^,^^23^ Since PCa does not show clear visible boundaries with respect to normal prostate tissues and nonglandular tissues are mixed with PCa, accurately extracting glandular epithelial (GE) tissue is the main problem in transcriptomic analysis on PCa. Advanced sequencing technologies, such as single-cell analysis^24^^,^^25^^,^^26^^,^^27^^,^^28^^,^^29^ and spatial transcriptomics (ST),^8^^,^^30^^,^^31^ have been used to detect transdifferentiation drivers in different malignant stages of PCa. Single-cell analysis can support transcriptome analysis at a cellular resolution. However, this approach lacks spatial information and can be circumvented by bioinformatics inference to some extent.^32^^,^^33^ Conventional ST combines the advantages of traditional histological technique with the high throughput of RNA sequencing, reflects entire transcriptomes at spatial dimensions^34^ and overcomes the limitation of quantitative gene *in situ* sequencing techniques.^35^^,^^36^ Nevertheless, the low resolution of spatial microarrays astonishingly allows 10–100 cells to be included in each spot, making it difficult to precisely determine the histological structures and cell features.^30^

Therefore, ST analysis at a higher spatial resolution is needed to maximally reflect individual cells. The Visium Spatial Gene Expression Solution provides spatial microarrays in which each spot has a 55 μm diameter, which allows the maximal distinction of histological structures. Hence, we conducted this study using Visium ST analysis, comprehensively analyzed the transcriptome-wide expression patterns of GE cells with different GS scores and inferred the crucial molecular events and associated biological functions during PCa progression.

## Results

### Flow path of Visium spatial transcriptomics analysis in prostate cancer tissues

**Figure 1:**
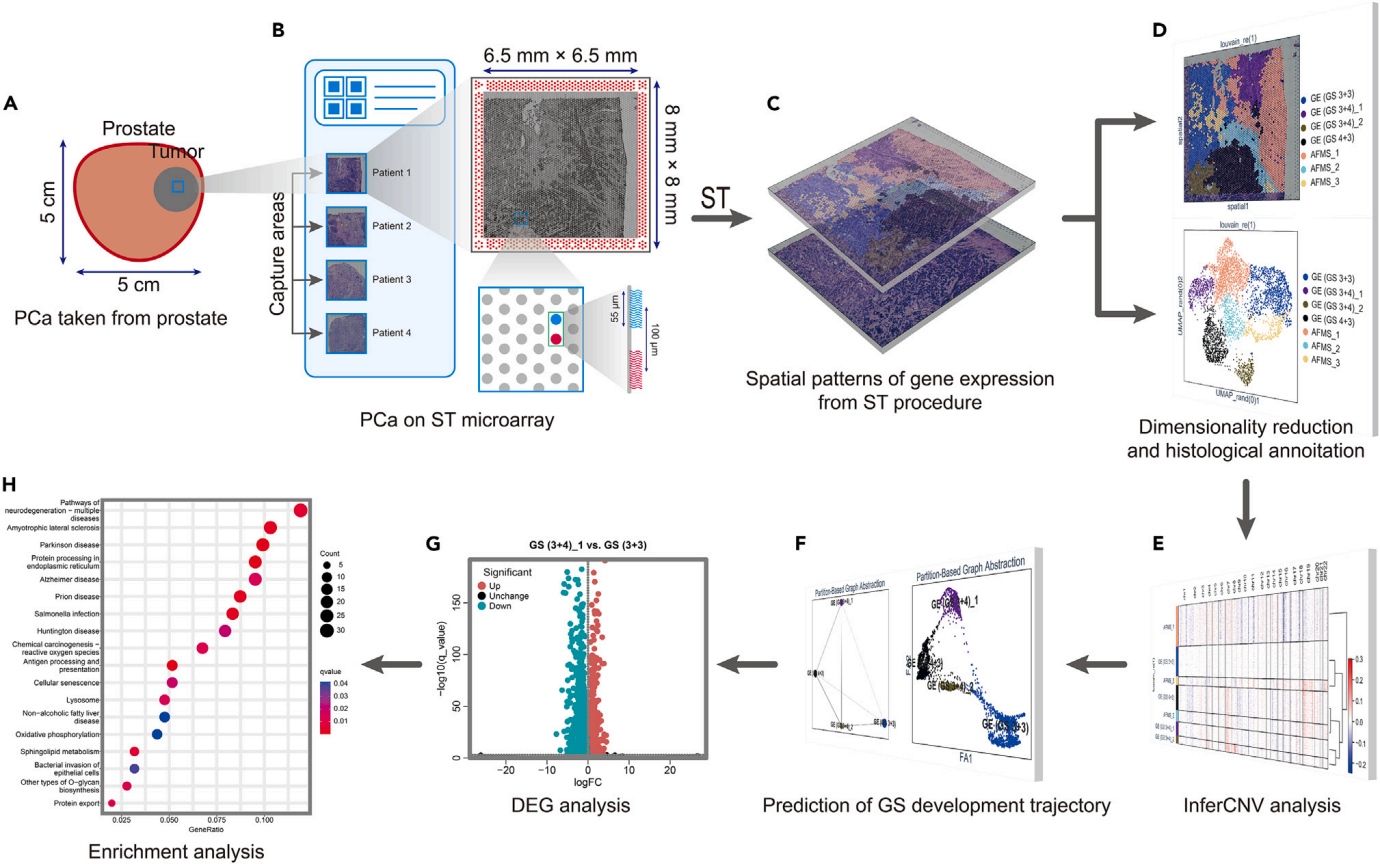
Flow path of Visium ST in PCa (A) Four PCa tissues were removed from four patients based on the area of biopsy results. (B) The qualified tissues were placed on Visium Spatial slides, which included 4992 spatially barcoded spots with a 55 μm diameter and a 100 μm center-to-center distance. The fiducial frame (red spots) was printed as a border around the capture areas to maintain orientation. (C) ST analysis was performed, and the matrices of read counts for whole genes in every spot were obtained. (D–H) Bioinformatics methods such as dimensionality reduction (D), inferCNV analysis (E), prediction of GS development trajectory via DPT and PAGA analyses (F), DEG (G), and enrichment analyses such as GO and KEGG (E) were performed to explore a set of crucial genes and biological functions related to PCa progression.

The workflow of this study is shown in Figure 1. Four separated PCa tissues were taken from four patients who underwent radical prostatectomy (Figure 1A). Information on the clinicopathological characteristics of the patients is shown in Table 1. We performed Visium ST (10× Genomics, Pleasanton, CA, USA) to analyze the spatial landscape of gene expression. The spatial microarrays had 4992 unique barcoded spots (with a diameter of 55 μm and center-to-center distance of 100 μm) with capture areas of 6.5 × 6.5 mm^2^ (Figure 1B). Each spot contained transcriptome-wide information for whole genes. We obtained the matrices of read counts and then transformed them to transcripts per kilobase of exome per million (TPM) mapped reads (Figure 1C). After dimensionality reduction via principal component analysis (PCA), the clusters of the spots were differently colored and annotated with different pathological types (such as different Gleason scores (GSs) of glandular epithelial (GE) tissues or anterior fibromuscular stroma (AFMS) tissues) in accordance with the orientation of the histological image (Figure 1D). Further bioinformatics methods, such as inferred copy number variation (inferCNV), diffusion pseudotime (DPT) and partition-based graph abstraction (PAGA) analyses, differentially expressed gene (DEG) analysis, and enrichment analysis (Figures 1E–1H), were performed to explore a set of crucial factors (genes and biological functions) related to PCa progression.

**Table 1:** Clinicopathological characteristics of selected patients with PCa

Patient number	Age	TPSA (ng/mL)	MRI (PI-RADS)	Endocrinotherapy	GS (biopsy)	GS (pathology)	Size range (cm)	IHC
Patient 1	65	23.91	None	None	3 + 3∼3 + 4	3 + 4	4.8 × 2.5×2.3	P504s (+), P63 (−), 34βE12 (−)
Patient 2	71	32.4	5	None	4 + 3–4 + 5	4 + 4	3.2 × 3×1.5	P504s (+), P63 (−), 34βE12 (−)
Patient 3	63	28.87	3∼4	None	4 + 3–4 + 4	5 + 4	2.7 × 2×1.1	P504s (+), P63 (−), 34βE12 (−)
Patient 4	72	9.86	None	Triptorelin pamoate (15 mg, twice)	3 + 3	4 + 3	Bilateral glands	P504s (+), P63 (−), 34βE12 (−)

TPSA, total prostate specific antigen; MRI, magnetic resonance imaging; PI-RADS, prostate imaging-reporting and data system; GS, Gleason score; IHC, immunohistochemistry.

### Transcriptomic and genomic heterogeneity of prostate cancer tissue from patient 1

**Figure 2:**
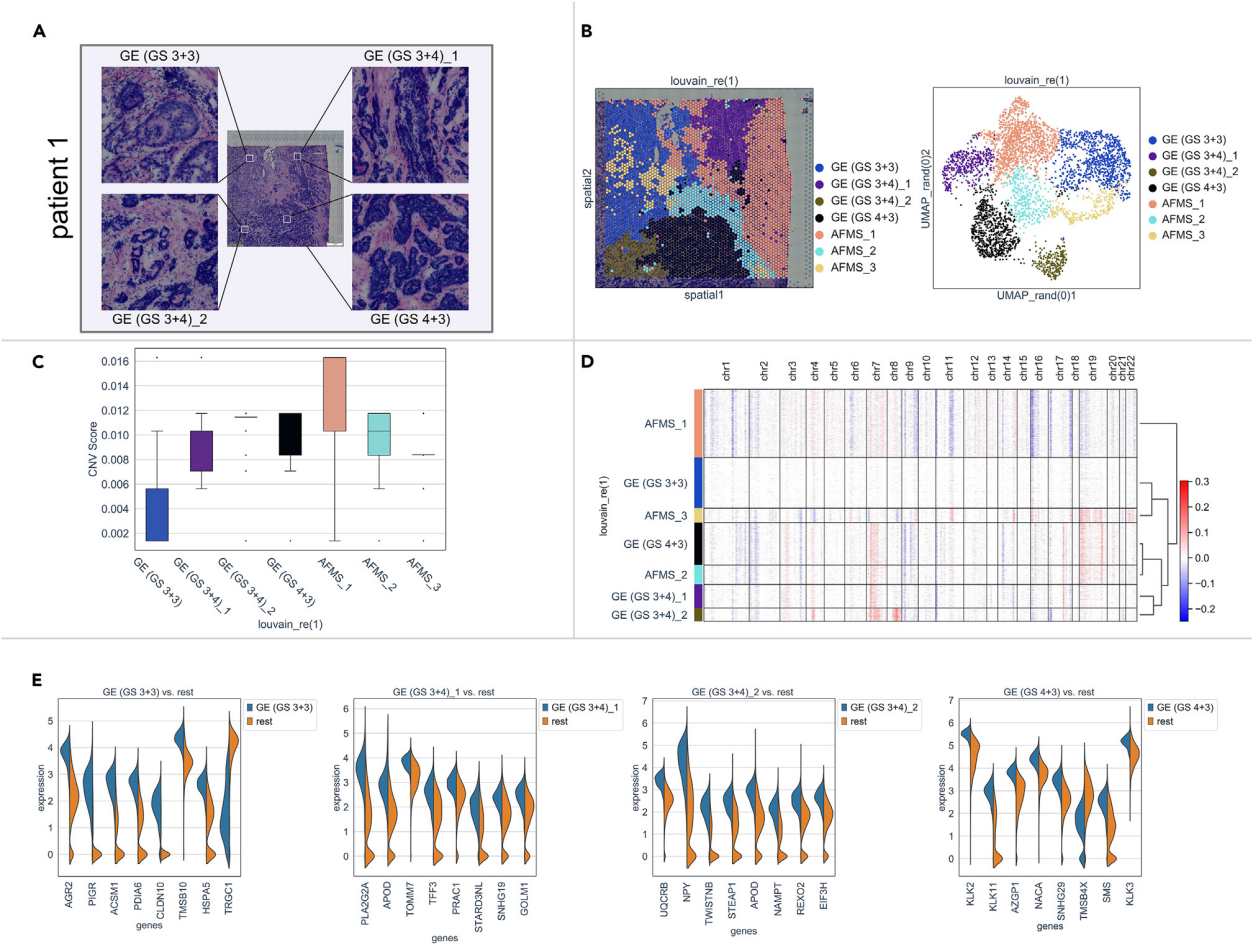
Transcriptomic and genomic heterogeneity of PCa tissue from patient 1 (A) H&E-stained image of PCa from patient 1. Four different histological structures of lesions are enlarged and annotated with GSs. (B) The genetic classifications across all spots were analyzed via PCA. The spots of the subclusters overlaid on the histological images were annotated with GSs and AFMS on the basis of histological structures (left) and were visualized by UMAP (right). (C) The CNV value of each gene was calculated by inferCNV, and the average CNV score of each subgroup is shown in a histogram. (D) The chromosomal landscape of inferCNVs distinguishing subgroups of GEs and AFMS is shown in a heatmap. The horizontal axis represents chr1 to chr22, the left vertical axis represents the spot subclusters, and the right vertical axis represents unsupervised hierarchical clustering. Red: gain of copies, blue: loss of copies. (E) DEGs in paired comparisons between each individual GS and the others are shown in violin plots. The x axis represents the 8 genes with the lowest Q-values in Wilcoxon test, and the y axis represents the expression levels of each individual GE subcluster (left half) and the others (right half) in the violin plot.

Multifocal PCa is reported to show GS heterogeneity.^37^ PCa tissue from patient 1, whose GSs according to needle biopsy and postoperative pathology were both 3 + 4 = 7, was initially analyzed (Table 1). ST analysis showed that EEF1A1, KLK2, KLK3, and MALAT1 were the most highly expressed genes in this sample (Figure S1A). In the hematoxylin-eosin (H&E)-stained image, we annotated the spatial patterns of GSs among different lesions (Figure 2A). In accordance with the pathological information, there were histologically identifiable structures of GS 3 + 3, 3 + 4, and 4 + 3 in GE cells (annotated as GE (GS 3 + 3), GE (GS 3 + 4)_1, GE (GS 3 + 4)_2, and GE (GS 4 + 3)) of this section (Figure 2A). The transcriptomic classifications across all spots in the PCa section were explored via PCA and visualized by UMAP (Figure 2B). The clustering spots overlaid on the histological image generally exhibited closely mirrored spatial patterns of the histological structures of GSs and AFMS areas (Figure 2B). We annotated these subgroups and found that the GE (GS 3 + 3) was well differentiated and showed low-grade cellular changes, with the most similar appearance to normal tissues. We then performed inferCNV analysis with GE (GS 3 + 3) as the reference group to predict clonal hierarchies and tumor malignancy.^4^^,^^5^^,^^6^^,^^7^^,^^8^^,^^25^^,^^26^ The average copy number variation (CNV) value was estimated as the basis for identifying the malignancy of the histological clusters.^25^^,^^26^ We found stronger CNV scores in the GE (GS 4 + 3) and GE (GS 3 + 4)_2 groups than in the GE (GS 3 + 4)_1 and GE (GS 3 + 3) groups (Figure 2C). In the unsupervised hierarchical clustering analysis using the CNV values of the chromosomal landscape, two GS 3 + 4 groups, GE (GS 3 + 4)_1 and GE (GS 3 + 4)_2, were clustered together, adjacent to GE (GS 4 + 3) (Figure 2D), implying their similarity and monoclonal origin (Figure 2D). All spots were divided into 10 clusters (CNV_1 to CNV_10) via PCA with CNV values (Figure S2A). The pattern of the components of histological subgroups exhibited no obvious relationship with the CNV cluster pattern (Figure S2B).

DEGs in paired comparisons between each individual GS and the others were analyzed, and the 8 most significant genes (ordered according to increasing Q-values in Wilcoxon-test) are shown in Figure 2E. Considering the log fold changes (FCs), we extracted the representative factors PIGR, PLA2G2A, TWISTNB, and KLK11, which are generally expressed in the histological clusters of GE (GS 3 + 3), GE (GS 3 + 4) _1, GE (GS 3 + 4)_2, and GE (GS 4 + 3), respectively (Figures S2C and S2D).

### Spatial gene expression patterns during Gleason scores progression in patient 1

**Figure 3:**
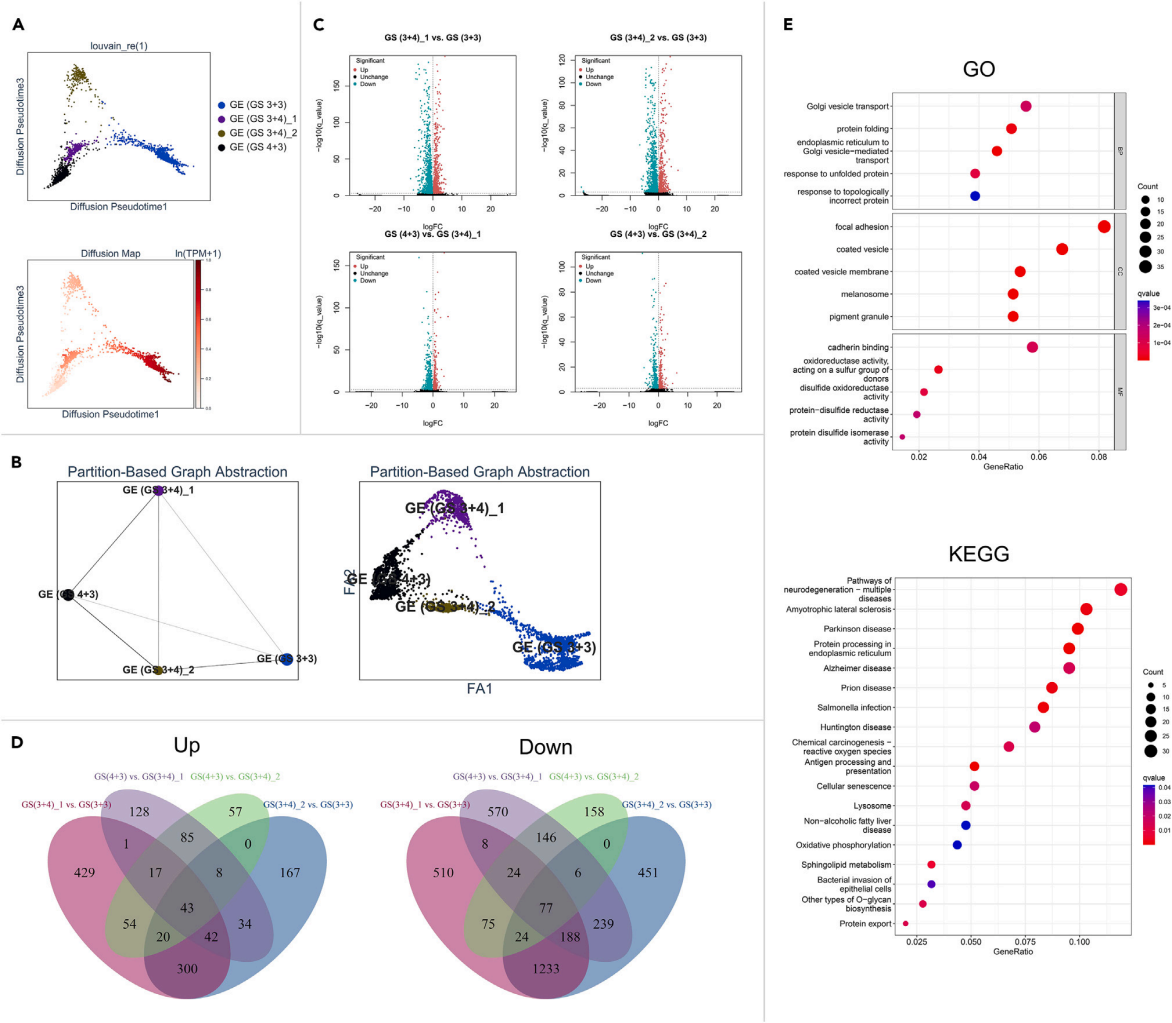
Spatial gene expression patterns during GS progression in patient 1 (A) The developmental paths of different GSs (top) were inferred through DPT analysis. The diffusion map (down) was used to construct appropriate coordinates. (B) The inference of the GS development trajectory on the basis of retaining the GE subclusters (left) and each spot (right) map was performed via a static PAGA plot. (C) The DEGs in the comparisons among GE clusters of GS (3 + 4)_1 vs. GS (3 + 3), GS (3 + 4)_2 vs. GS (3 + 3), GS (4 + 3) vs. GS (3 + 4)_1, and GS (4 + 3) vs. GS (3 + 4)_2 were analyzed, and the significantly altered genes (Q-value <0.001 in Wilcoxon-test) were visualized in Volcano plots. Red spots: high expression, green spots: low expression, black spots: no significant difference in the comparison. (D) The simultaneously upregulated (left) or downregulated (right) genes in the four comparisons are summarized and shown in a Venn diagram. (E) The results of GO (up) and KEGG (down) enrichment analyses using simultaneously dysregulated genes among the ≥3 of the 4 comparisons are shown in dot plots. BP, biological processes; CC, cellular components; MF, molecular function.

Based on the transcriptome-wide data, the developmental trajectories of GSs in patient 1 were inferred by determining geodesic distances along the graph. According to the DPT and PAGA results, the GE (GS 4 + 3) cluster was near GE (GS 3 + 4)_1 and GE (GS 3 + 4)_2, which were separated from GE (GS 3 + 3) (Figures 3A and 3B). These findings were in accord with the idea that PCa with GS ≥ 7 should be identified as highly aggressive disease.^18^^,^^19^

We then sought to identify different biological functions during GS development. To investigate the GS progression-associated genes, we analyzed the DEGs among the comparisons of GS (3 + 4)_1 versus (vs.) GS (3 + 3), GS (3 + 4)_2 vs. GS (3 + 3), GS (4 + 3) vs. GS (3 + 4)_1, and GS (4 + 3) vs. GS (3 + 4)_2 (Figure 3C). Forty-three genes including KLK2 and KLK11 were simultaneously upregulated, and seventy-seven genes including CD46, CD59, and F5 were simultaneously downregulated in the 4 comparisons (Figure 3D). The intersecting (130 upregulated and 319 downregulated) genes among ≥3 of the 4 comparisons were used for enrichment analysis. The biological functions of Golgi vesicle transport, focal adhesion, and cadherin binding in Gene Ontology (GO) analysis (Figure 3E) and neurodegeneration in Kyoto Encyclopedia of Genes and Genomes (KEGG) analysis were the most enriched during GS progression in patient 1 (Figure 3E).

To verify these GS development-related genes, we used the prostate cancer of The Cancer Genome Atlas (TCGA-PRAD) dataset and evaluated the expression levels of 117 intersecting genes (among the 120 simultaneously dysregulated genes, the expression of 1 excluded gene, TOMM6, was 0, and 2 excluded genes, AP004608.1 and H3F3A, were not identified in TCGA-PRAD dataset) associated with various clinicopathological characteristics. We found that the expression levels of 82 genes (including KLK2 and HNRNPC) significantly differed between PCa and normal prostate tissues (Figure S3A; Table S1), and those of 45 and 46 genes (including KLK2, NANS, and CRIP2) significantly differed when classifying patients according to GS grade (GS > 7 compared with GS < 7) and pathological T (pT) stage (pT3 compared with pT2) (Figures S3B and S3C; Tables S2 and S3) (p < 0.05). TP53 mutation is a crucial criterion for identifying PCa malignancy and indicates a shorter time to CRPC and radiographic progression.^38^ Thirty-five genes were differentially expressed between the TP53 mutation group and the wild-type group (Table S4).

Survival analysis was performed to evaluate the effects of 116 genes (ADIRF could not identify cutoff values and was thus excluded) on the prognostic outcomes of TCGA-PRAD. The 495 patients were divided into two groups according to the optimum threshold segmentation of gene expression (with the lowest log rank P-value in the Kaplan−Meier analysis). We found that 16 and 20 genes (including NANS, CRIP2, and CNIH4) significantly influenced the recurrence-free survival (RFS) and progression-free survival (PFS) outcomes, respectively, 9 and 13 of which were associated with poor prognosis (HR > 1 and P-value <0.05 in the Cox regression analysis) (Figures S3D and S3E; Tables S5 and S6).

**Figure 4:**
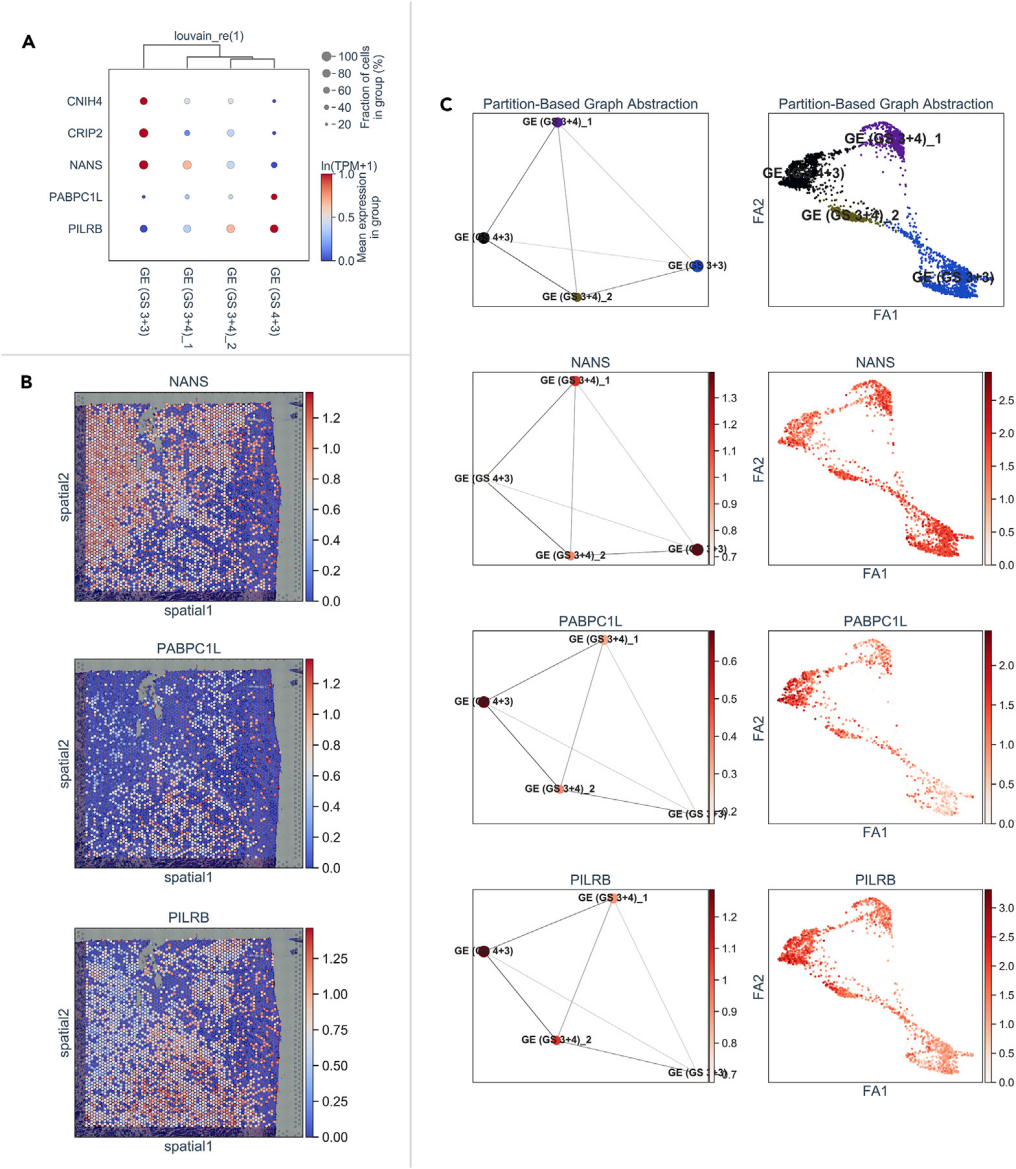
Expression patterns of simultaneously altered genes in the TCGA-PRAD dataset and ST analysis in patient 1 (A) The expression patterns of the 5 intersecting genes in the comparisons of various clinicopathological characteristics and prognoses of the TCGA-PRAD dataset are shown in a factor bubble plot. The x axis represents four GE subclusters, and the y axis represents 5 intersecting genes. Red: high expression, blue: low expression. (B) The expression levels of the 3 representative genes simultaneously altered in the TCGA-PRAD dataset and ST analysis are shown in spatial activity maps in the histological image. (C) The expression levels and distribution of the 3 genes on the basis of retaining the GE subclusters (left) and each spot (right) map in PAGA plots of four GE subclusters.

We summarized 5 intersecting genes, CNIH4, CRIP2, NANS, PABPC1L, and PILRB, that were simultaneously dysregulated in the comparisons of the PCa vs. normal, GS > 7 vs. GS < 7, pT3 vs. pT2 groups, and the survival analysis in RFS and PFS (p < 0.05) (Figures S3F and 4A). However, diverse expression tendencies of these genes were found between the ST analysis and the TCGA-PRAD dataset under relatively advanced-stage parameters. For example, CNIH4 and CRIP2 acted as inhibitors of GS progression in the ST analysis, but the expression of both genes was upregulated in the most advanced stages of TCGA-PRAD (in the GS > 7, pT3, TP53 mutation, and HR > 1 in survival analysis of RFS and PFS) (Table S7). This disagreement may be caused by the intratumor heterogeneity and histological atypia of the extracted lesions from multifocal PCa, leading to discordance between the gene expression results and the actual pathological diagnosis in the TCGA-PRAD dataset. However, the expression of genes, such as NANS, PABPC1L, and PILRB, exhibited a generally consistent trend toward advanced stages in the ST analysis and the TCGA-PRAD dataset (Table S7). The spatial activity maps and the expression patterns of the 3 genes in the PAGA graph indicate the GS progression patterns (Figures 4B and 4C).

### Spatial transcriptome and genomic heterogeneity and patterns during Gleason scores progression in patient 2

**Figure 5:**
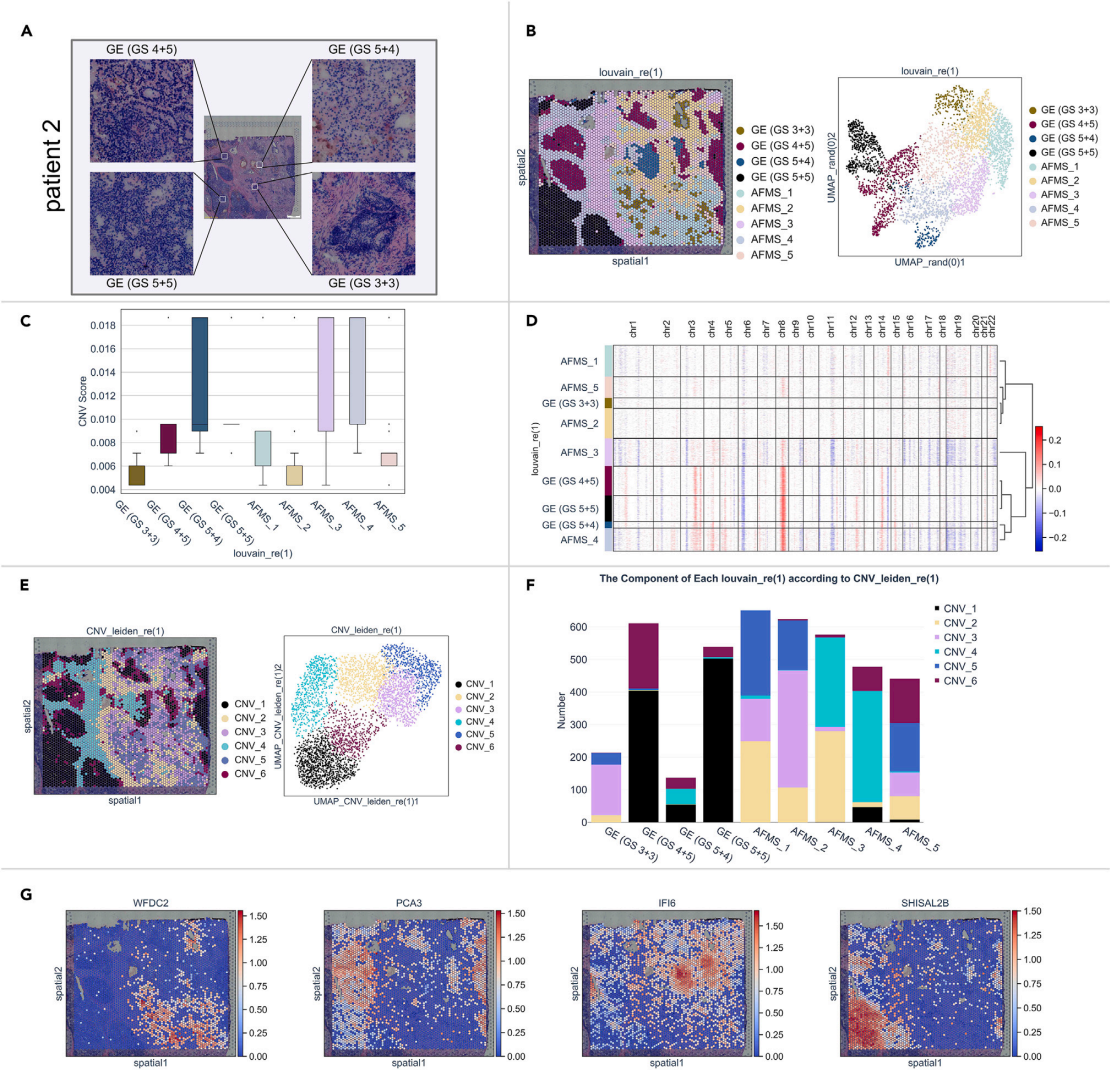
Transcriptomic and genomic heterogeneity of PCa tissue from patient 2 (A) H&E-stained image of PCa from patient 2. Four different histological structures of lesions are enlarged and annotated with GSs. (B) The genetic classifications across all spots were analyzed via PCA. The spots of the subclusters overlaid on the histological image were annotated with GSs and AFMS on the basis of histological structures (left) and visualized by UMAP (right). (C) The CNV value of each gene was calculated by inferCNV, and the average CNV score of each subgroup is shown in a histogram. (D) The chromosomal landscape of inferCNVs distinguishing subgroups of GEs and AFMS is shown in a heatmap. The horizontal axis represents chr1 to chr22, the left vertical axis represents the spot subclusters, and the right vertical axis represents unsupervised hierarchical clustering. Red: gain of copies, blue: loss of copies. (E) The classifications across all the spots on the basis of the CNV values were determined via PCA. The spots of the subclusters overlaid on the histological image were annotated with CNV_1 to CNV_6 (left) and visualized by UMAP (right). (F) The components of histological subgroups according to CNV clusters are shown in histograms. The x axis represents histological subgroups, and the y axis represents the number of spots in CNV clusters. (G) The expression levels of the crucial factors that are generally expressed in the different GSs are shown in spatial activity maps in the histological image.

The GSs of patient 2 were 4 + 3 to 4 + 5 according to needle biopsy and 4 + 4 according to postoperative pathology. ST sequencing of a section from this patient showed that KLK3, EEF1A1, FTL, TAGLN, and KLK2 were the most highly expressed genes (Figure S1B). Regions of GS 3 + 3, 4 + 5, 5 + 4, and 5 + 5 were observed in the H&E-stained image of this tissue (Figure 5A). The results of dimensionality reduction analysis still matched the results for classified spots with the histological structures of GSs and AFMS (Figure 5B). The average CNV values of classified regions gradually increased during GS upgrading, suggesting the consistency of PCa malignancy identified by CNV scores with GS stages (Figure 5C). Clustering analysis of CNV values showed that GE (GS 4 + 5) and GE (GS 5 + 5) were clustered together, adjacent to GE (GS 5 + 4) (Figure 5D). All the spots were further classified into 6 subgroups (CNV_1 to CNV_6) through the PCA of inferCNV (Figure 5E). The component analysis showed that CNV_1 was commonly included in the histological clusters of GE (GS 4 + 5), GE (GS 5 + 4), and GE (GS 5 + 5), implying the potential relationships of CNV_1 during GS development (Figure 5F).

Within the expression patterns, we analyzed the DEGs in paired comparisons between each individual GS cluster and the others and found that WFDC2, PCA3, IFI6, and SHISAL2B were specifically overexpressed factors in subgroups GE (GS 3 + 3), GE (GS 4 + 5), GE (GS 5 + 4), and GE (GS 5 + 5), respectively (Figures 5G, S4A, and S4B).

**Figure 6:**
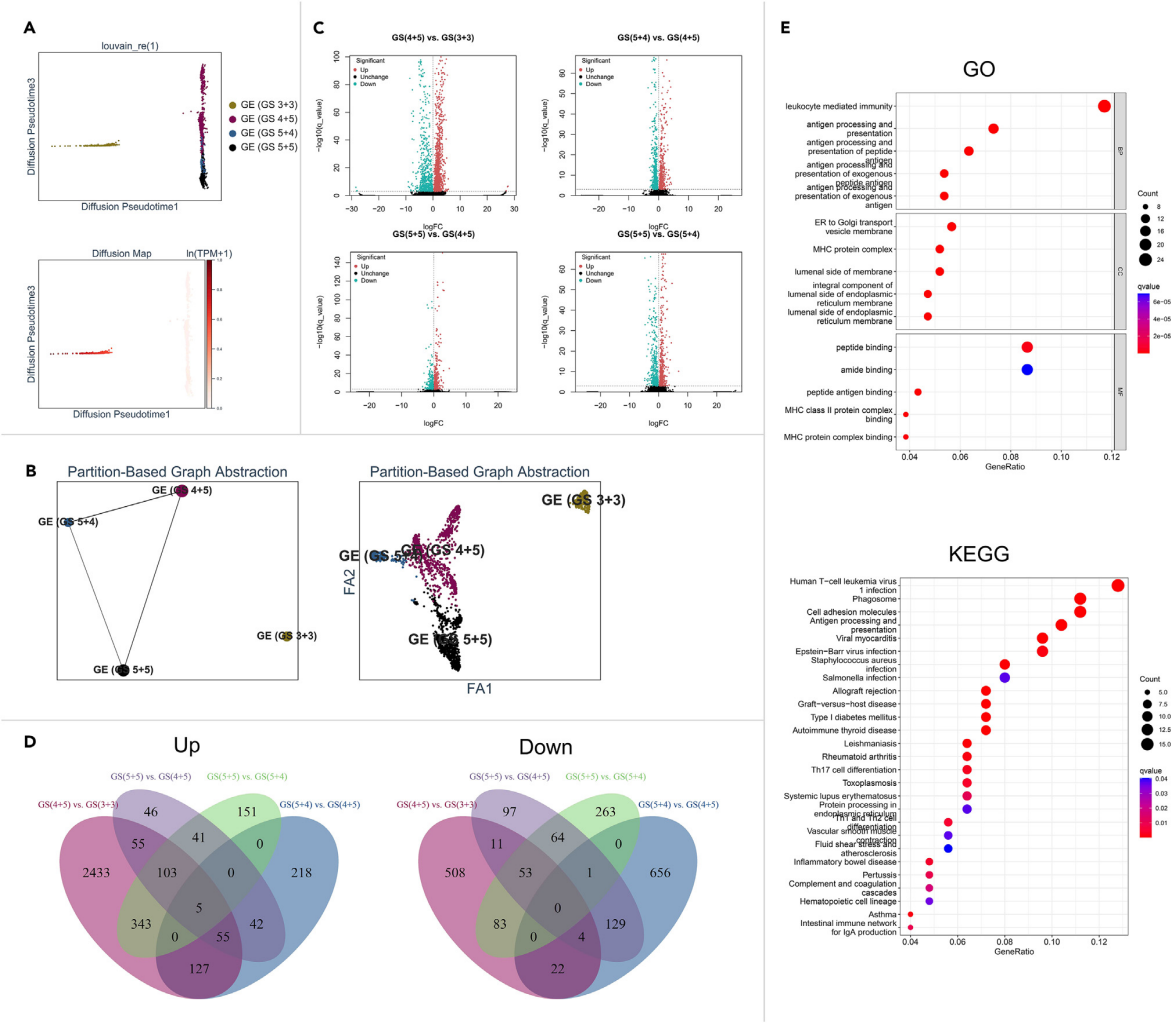
Spatial gene expression patterns during GS progression in patient 2 (A) The developmental paths of different GSs (top) were inferred through DPT analysis. The diffusion map (down) is used to construct appropriate coordinates. (B) The inference of the GS development trajectory on the basis of retaining the GE subclusters (left) and each spot (right) map was performed via a static PAGA plot. (C) The DEGs in the comparisons among GE clusters of GS (4 + 5) vs. GS (3 + 3), GS (5 + 4) vs. GS (4 + 5), GS (5 + 5) vs. GS (4 + 5), and GS (5 + 5) vs. GS (5 + 4) were analyzed, and the significantly altered genes (Q-value <0.001) were visualized in Volcano plots. Red spots: high expression, green spots: low expression, black spots: not significant between the comparison. (D) The simultaneously upregulated (left) or downregulated (right) genes in the four comparisons are summarized and shown in a Venn diagram. (E) The GO (up) and KEGG (down) enrichment analyses using simultaneously dysregulated genes among the ≥3 of the 4 comparisons are shown in dot plots.

Close relationships and developmental trajectories were observed among the histological clusters of GE (GS 4 + 5), GE (GS 5 + 4), and GE (GS 5 + 5) in the DPT and PAGA analyses (Figures 6A and 6B). However, the GE (GS 3 + 3) cluster was distinct from and uncorrelated with the others, which may be the reason that it was well differentiated and closer to the normal GE cells (Figures 6A and 6B).

We identified dysregulated factors associated with GS progression among the comparisons of GS (4 + 5) vs. GS (3 + 3), GS (5 + 4) vs. GS (4 + 5), GS (5 + 5) vs. GS (4 + 5), and GS (5 + 5) vs. GS (5 + 4) (Figure 6C). Five DEGs, ASAH1, MIR99AHG, PART1, PPFIA2, and SESN3, were simultaneously overexpressed in the 4 comparisons (Figure 6D). In the enrichment analyses using intersecting genes among ≥3 of the 4 comparisons, leukocyte-mediated immunity was enriched in the GO analysis, and human T cell leukemia virus 1 infection was enriched in the KEGG analysis (Figure 6E).

We then verified these genes using the TCGA-PRAD database mentioned in the description of patient 1 (Figures S5A–S5G; Tables S8–S13). The GS promoters in the ST analysis, PPFIA2 and SESN3, were upregulated in most advanced stages of TCGA-PRAD (in the GS > 7, pT3, TP53 mutation, and HR > 1 in survival analysis) (Figures S6A–S6C; Table S14).

### Spatial gene expression patterns of patient 3 and patient 4

In patient 3, the pathological diagnosis of the preoperative needle biopsy GS was 4 + 3 to 4 + 4, and the postoperative pathology was 5 + 4, with a size range of 2.7 × 2 × 1.1 cm. However, the vast majority of GE structures exhibited GS3, and only a few showed GS4 in the H&E-stained images of extracted tissue (Figure S7A). This serious discrepancy may have been caused by the multifocal PCa tissue and the difficulty of visually distinguishing histological structures, which potentially explains the discordance in gene expression between the TCGA-PRAD dataset and ST analysis for patient 1. ST sequencing showed that IGKC, IGLC2, EEF1A1, FTL, and PTGDS were the most highly expressed genes (Figure S1C). The PCA and UMAP analysis divided PCa into two GE clusters that approximately matched the histological structures of GS (3 + 3) and GS (3 + 4) (Figure S7B). Because of similar expression patterns, it was also difficult to identify specific expression patterns in GE (GS 3 + 4) and GE (GS 3 + 3) (Figures S7C–S7E). We found one cluster composed of cells such as those of inflammatory infiltrates and highly expressed immune-related genes (including CD74, TMSB10, HLA-DRB1, HLA-DRA, and HLA-DPB1), so we annotated this cluster as immune-related cell (IRC) (Figure S7C). InferCNV analysis revealed no significant pattern in the average CNV values, CNV clusters, or component analysis results between the two groups (Figures S8A–S8D). DPT and PAGA analysis indicated the relationship of the two clusters (Figures S9A and S9B). DEGs of GS 3 + 3 and GS 3 + 4 (Q-value <0.001 in Wilcoxon-test) were used for enrichment analysis (Figure S9C). The biological function terms regulation of protein transport, cell-substrate junction, and cadherin binding were the most enriched in the GO analysis (Figure S9D). Our previous study showed that the transmembrane protein N-cadherin could promote PCa progression via the N-cadherin/c-Jun/NDRG1 axis.^39^ Adherens junction was enriched during GS 3 + 3 to 3 + 4 progression in the KEGG analysis (Figure S9D).

Patient 4 underwent two rounds of endocrinotherapy with triptorelin pamoate (15 mg per 3 months), and the postoperative pathology showed a GS of 4 + 3. In the H&E-stained image, we found that GE cells occupied the entire tissue, and all of them were categorized as GS 4 + 3 (Figure S10A). Although the tissue was divided into 8 clusters via PCA, no difference in the histological structure was found among the clusters, so it was difficult to estimate the evolutionary pattern (Figure S10B). EEF1A1, FTL, KLK3, KLK2, and MALAT1 were the top 5 expressed genes (Figure S1D). Within the expression patterns, we found that immediate-early response proto-oncogenes, such as FOS, JUN, FOSB, and JUNB, were significantly overexpressed in GS (GE 4 + 3)_7 (Figures S10C–S10E).^40^

### Simultaneously dysregulated genes in patients 1–3 during Gleason scores progression

**Figure 7:**
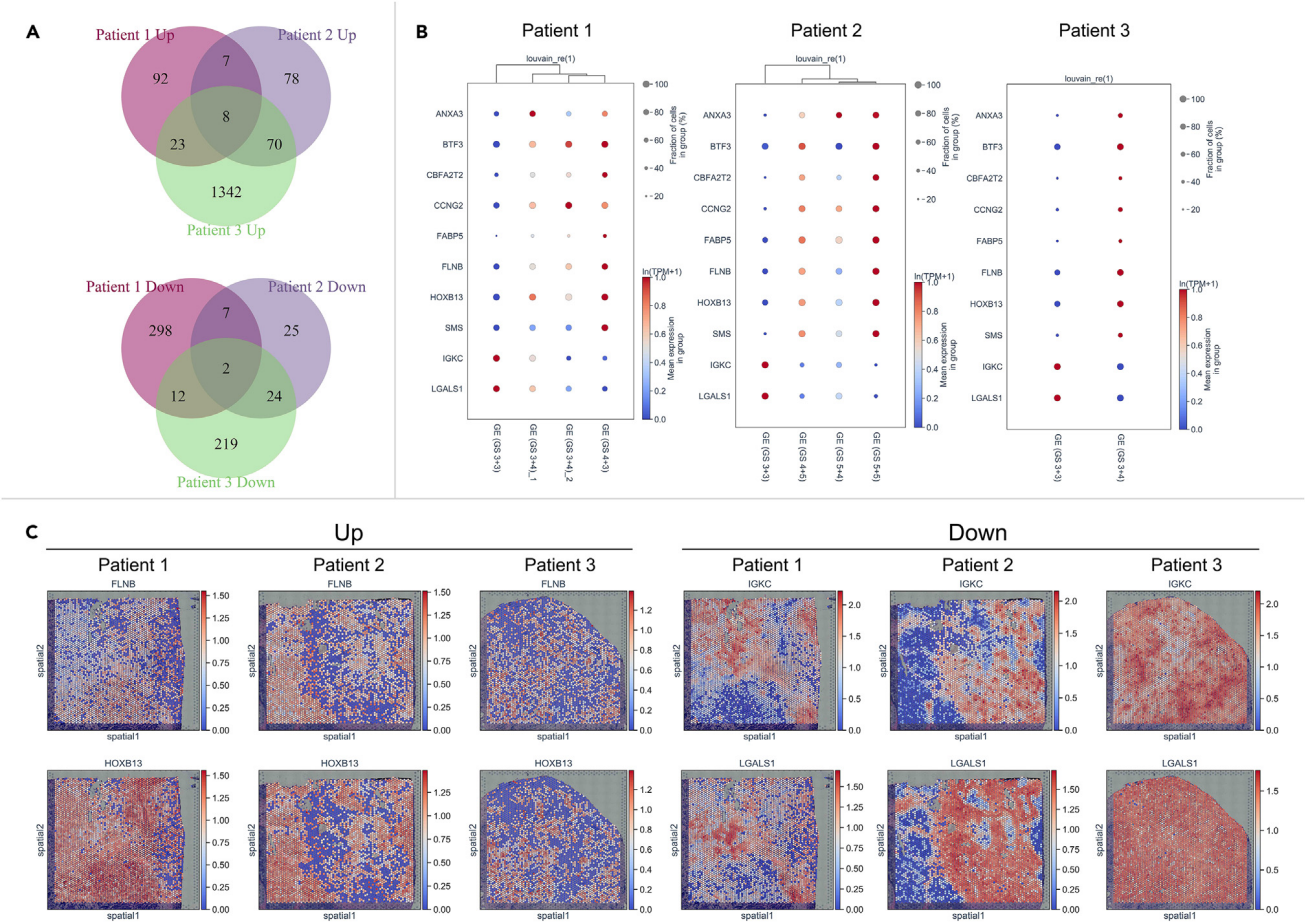
Simultaneously dysregulated genes during GS development in patients 1–3 (A) Simultaneously, upregulated (up) or downregulated (down) genes among ≥3 of the 4 different GE comparisons in patients 1–2 and in the GE comparison of patient 3 mentioned above are illustrated in Venn diagrams. (B) The expression patterns of the 10 intersecting genes indicated above in the GEs of patients 1–3 (from left to right) are shown in a bubble plot. The x axis represents four GE subclusters, and the y axis represents 10 intersecting genes. Red: high expression, blue: low expression. (C) The expression levels of the representative simultaneously upregulated genes FLNB and HOXB13 (left) and downregulated genes IGKC and LGALS1 (right) are shown in spatial activity maps in the histological image.

We finally analyzed the simultaneously upregulated or downregulated genes during GS development in patients 1–3. The intersecting genes (Q-value <0.001 among ≥3 of the 4 comparisons in patients 1–2 and Q-value <0.001 in the comparison of patient 3 for the different GSs mentioned above) were analyzed, and 10 of them (upregulated genes of ANXA3, BTF3, CBFA2T2, CCNG2, FABP5, FLNB, HOXB13, and SMS, and downregulated genes of IGKC and LGALS1) were simultaneously dysregulated, suggesting that most genes have individual specificity during GS upgrading (Figures 7A and 7B). The activities of the GS promoters, FLNB and HOXB13, and the GS inhibitors, IGKC and LGALS1, are shown in spatial maps and a PAGA graph (Figures 7C and S11A–S11C).

## Discussion

The prostate is mainly composed of GE and nonglandular (AFMS) tissues, and PCa is initiated from luminal or basal cells of the GE.^20^^,^^21^^,^^22^^,^^23^ In contrast to other urothelial cancers, PCa is not characterized by an envelope and is instead diffusely distributed with multiple foci. The PCa sections removed during our visual assessment did not show a clear boundary with respect to normal tissue and were mixed with nonglandular tissues, causing confusion in the pathological diagnosis. Therefore, conventional transcriptional sequencing analysis cannot accurately reveal the genetic patterns in PCa cells. The serious discrepancy of histological GS assessment in patient 3 and the discordance between ST analysis and the TCGA-PRAD dataset regarding the gene expression patterns of patients 1–2 confirmed this point.

Visium ST technology quantifies an array of transcriptomes and provides a high spatial resolution across PCa sections, allowing the precise analysis of transcriptome-wide heterogeneity in GE cells. The accuracy of the ST-based assessment of spatial gene activity was confirmed in a previous study by corresponding protein staining through immunohistochemical (IHC).^30^ To ensure that the extracted PCa sections were appropriate for the analysis, we sampled more than 30 patients with PCa and assessed RNA quality and histological structures based on integrity number (RIN) values, H&E, and IHC staining. Finally, we chose the most representative 4 tissues with GS development patterns, which were 3 + 3 to 3 + 4 (Patient 3), 3 + 3 to 4 + 3 (Patient 1), 3 + 3 to 5 + 5 (Patient 2), and 4 + 3 with endocrinotherapy (Patient 4), and comprehensively analyzed dysregulated genes and biological functions during GS progression.

To analyze the evolutionary process of PCa, we need to understand the relationship and origin of multiple lesions within one sample. This is still controversial, and there are currently two main acknowledged theories: independent initial multiclonality^41^^,^^42^^,^^43^^,^^44^ and evolution from a monoclonal disease.^4^^,^^5^^,^^6^^,^^7^^,^^8^ The first theory is rooted in the patterns of allelic imbalances^42^^,^^43^^,^^44^ and single nucleotide variants (SNVs).^41^ Although this theory was previously accepted, the relevant studies were restricted to considering a limited number of prevalent somatic aberrations as the dominant clone, and they could not exclude the possibility that underlying subclonally maintained common mutations were missed by the underpowered method. Variable patterns of genomic aberrations arise during the development of advanced stages, distant metastases, and therapeutic resistance in PCa.^4^^,^^5^^,^^6^^,^^7^^,^^8^^,^^45^^,^^46^ This acquired genomic instability triggers evolutionary processes to form distinct multiple phenotypes of subclones. Therefore, several authoritative studies currently support the second theory, that multifocal PCa emerges from a monoclonal origin,^4^^,^^5^^,^^6^^,^^7^^,^^8^ with an ongoing Darwinian evolutionary process.^47^^,^^48^^,^^49^ Using high-resolution genome-wide analysis, it was found that the cancer cells from which multiple PCa foci originate in a single case could be traced to the same allele-specific copy-number changes, which stably replicated during cell division.^4^^,^^5^^,^^8^

This study is based on the theory that intratumor GS heterogeneities represent the diverse progression status evolved from a single PCa precursor. A previous study confirmed that the factor activity maps obtained in ST analysis generally show spatial patterns closely mirroring histologically identifiable structures, and further classification by PCA clearly separates these structures into, for example, PCa with GS 3 + 3, normal glands, and prostatic intraepithelial neoplasia (PIN) glands.^30^ We classified all the spots in PCa sections into subgroups by PCA and found that the subgroups overlaid on the histological images generally matched the histological structures of different GSs. We then inferred the factors corresponding to each GE via comparison with the remaining subgroups and combined them with spatial activity patterns. Well-known PCa-related genes, such as KLK3, KLK2, NPY, PCA3, and ACPP, exhibited a high frequency in association with the GSs of different patients. However, we observed that the same GSs from different patients or subgroups were associated with different generally expressed genes, implying individual heterogeneity during PCa progression.

We analyzed the origin and malignancy of each histological structure through inferCNV analysis. InferCNV requires a reference value, where “normal” GE cells are needed for studies of the comparison of expression among genomic regions. However, a statistical problem arose because our PCa tissues did not contain normal prostate tissue. The current report proposes that GS (3 + 3) represents a precancerous lesion^50^ because of well-differentiated, low-grade cellular changes, and a metastasis-free nature.^18^ We chose GS (3 + 3) as a reference in patients 1–3. To verify the CNV values, we next assessed PCa malignancy based on CNV scores in different subgroups and revealed a tendency consistent with the GS evaluation results. Further clustering of the inferCNV results revealed that the GS groups (except for the reference group) of different patients clustered together, implying similar CNV changes and a monoclonal origin of different GSs.

The developmental trajectories of different histological structures were evaluated through DPT and PAGA analyses. We found structures with GS ≥ 7 in one PCa section that were near each other and exhibited developing trajectories during GS upgrading. However, GS (3 + 3) in patient 1 and patient 2 was distinct from the other aggressive PCa fractions, which were well differentiated and less malignant.

After the preliminary determination of a monoclonal origin and developmental trajectories in the different GS clusters, we discovered dysregulated biological functions during GS progression. The DEGs identified in the pairwise comparisons according to GS progression were screened, and the intersecting genes were used for further enrichment analysis. We found that the DEGs and related biological functions varied in different patients, suggesting individual heterogeneity of PCa. The biological functions such as cadherin binding, Golgi vesicle transport, and protein folding were associated during GS upgrading. Our previous study demonstrated that N-cadherin was upregulated in PCa with GS > 7 and could promote PCa progression via c-Jun/NDRG1 signaling.^39^

The screened genes were verified using the TCGA-PRAD dataset. Although we found that genes, such as NANS, PABPC1L, PILRB, PPFIA2, and SESN3, presented consistent trends in the GS groups of ST and were associated with clinicopathological parameters and prognosis in the TCGA-PRAD dataset, discordance in the expression patterns of other genes, such as CNIH4, CRIP2, and PART1, also existed. The reason for these discrepancies is not clear, and they may be caused by individual heterogeneity in PCa or unrepresentative extracted tissues from multifocal PCa.

We then summarized the simultaneously dysregulated genes during GS development in patents 1–3. Although genes, such as FLNB, HOXB13, IGKC, and LGALS1, were generally dysregulated in the three patients, most genes exhibited individual specificity during GS upgrading. This implies that PCa is highly heterogeneous and requires individualized treatment clinically. Powerful sequencing technology has delivered novel biomarkers specific to aggressive PCa and contributed to the revolution of personalized therapy. This is clinically exemplified by substantially higher frequencies of aberrations revealed in homologous recombination repair-related genes, such as BRCA1, BRCA2, and ATM, in metastatic castration-resistant prostate cancer (mCRPC),^46^ which were shown to be associated with therapeutic sensitivity to a PARP inhibitor (olaparib).^51^^,^^52^ However, after a period of treatment, PCa becomes drug resistant and progressive. Neuroendocrine prostate carcinoma (NEPC) is recognized by neuroendocrine markers such as chromogranin A (CgA), synaptophysin (Syn), and neuron-specific enolase (NSE). NEPC is the most lethal PCa with an extremely poor prognosis. Regardless of the limited therapeutic effect, platinum-based chemotherapy agents, such as cisplatin and carboplatin, are typically used to treat NEPC according to the protocol for small cell lung cancer. Immunotherapy against B7-H3 (CD276) has been widely reported as a potential therapeutic target in advanced PCa.^53^^,^^54^^,^^55^ However, immune checkpoint inhibitor monotherapies have largely been ineffective in PCa,^56^^,^^57^ arguably due to the potent immunosuppressive mechanisms.^29^^,^^56^^,^^58^ A study supported that multiple immune alterations in the cribriform PCa (invasive cribriform carcinoma (ICC) and intraductal carcinoma (IDC)) tumor microenvironment (TME) contribute to overall immunosuppression.^29^ Therefore, novel therapeutic targets are urgently needed through sequencing technologies, experimental analyses, and clinical trials to improve PCa personalized therapy.

### Conclusions

Overall, this study overcame the limitations of traditional sequencing analysis and accurately evaluated the transcriptome-wide levels of GE cells at a high spatial resolution through Visium ST technology. We comprehensively analyzed the gene expression patterns in different histological structures in a spatial context based on samples from four patients with PCa and revealed developmental trajectories during GS upgrading. DEGs, such as NANS, PABPC1L, PILRB, PPFIA2, and SESN3, and dysregulated biological functions, such as cadherin binding, Golgi vesicle transport, and protein folding, were related to GS progression in our patients with PCa, which has potential value for PCa malignant evaluation in clinical diagnosis. However, we also found individual heterogeneity in the transcription levels of different patients with PCa during GS progression, which implies that personalized targeted therapy will play an increasing role in the future clinical treatment of PCa. In summary, this study serves as a foundation for understanding ST-based GS evolution in PCa tissues and provides insight into GS progression-related DEGs and biological functions.

### Limitations of the study

There are several limitations that warrant further study. First, only four PCa tissues were used in this study, and more samples need to be analyzed for more reliable results. Second, the GS progression-related key genes and biological functions in this study were determined according to Visium ST technology combined with the TCGA-PRAD dataset, which has not been experimentally validated and clarified, so it could not provide an exact conclusion. Finally, PCa with endocrine therapy in our sample influences the histological morphology of GE cells that are difficult to distinguish through H&E staining, implying challenges in searching for CRPC progression through Visium ST technology in further studies.

## STAR★Methods

### Key resources table

**Table:** 

REAGENT or RESOURCE	SOURCE	IDENTIFIER
**Biological samples**		

PCa tissues	Beijing Tongren Hospital	N/A

**Critical commercial assays**		

Visium Spatial Gene Expression Slide & Reagent kit	10x Genomics	V10N30-034
Visium Spatial Tissue Optimization Slide & Reagent kit	10x Genomics	201012

**Software and algorithms**		

cellSens Dimension Software	Olympus	N/A
10x Genomics Visium library preparation protocol	10x Genomics	200100
Space Ranger	10x Genomics	N/A
OmniAnalyzer Pro	Abiosciences	N/A
SVD	OmniAnalyzer Pro	N/A
UMAP	OmniAnalyzer Pro	N/A
Seurat	OmniAnalyzer Pro	N/A
inferCNV	OmniAnalyzer Pro	N/A
DPT	OmniAnalyzer Pro	N/A
PAGA	OmniAnalyzer Pro	N/A
DEG	OmniAnalyzer Pro	N/A
R software (version 4.0.3)	the R Core Team and the R Foundation for Statistical Computing	https://www.r-project.org/
clusterProfiler	R package, G Yu et al.^59^^,^^60^	https://bioconductor.org/packages/release/bioc/html/clusterProfiler.html
enrichplot	R package	https://bioconductor.org/packages/release/bioc/html/enrichplot.html
limma	R package, Ritchie ME et al.^61^	https://bioconductor.org/packages/release/bioc/html/limma.html
reshape2	R package	https://cran.r-project.org/web/packages/reshape2/index.html
ggpurb	R package	https://cran.r-project.org/web/packages/ggpubr/index.html
survival	R package	https://cran.r-project.org/web/packages/survival/index.html
survminer	R package	https://cran.r-project.org/web/packages/survminer/index.html
VennDiagram	R package	https://cran.r-project.org/web/packages/VennDiagram/index.html
grid	R package	https://cran.r-project.org/web/packages/grid/index.html
futile.logger	R package	https://cran.r-project.org/web/packages/futile.logger/index.html
formatR	R package	https://cran.r-project.org/web/packages/formatR/index.html
Microsoft Excel 2019 software	Microsoft Corp.	N/A

**Deposited data**		

PCa in The Cancer Genome Atlas (TCGA)	the TCGA data portal	https://portal.gdc.cancer.gov/
PFS data	UCSC Xena website	https://xenabrowser.net/datapages/

### Resource availability

#### Lead contact

Further information and requests for resources and reagents should be directed to and will be fulfilled by the lead contact, Hao Ping (pinghaotrh@ccmu.edu.cn).

#### Materials availability

This study did not generate new unique reagents.

#### Data and code availability

(1) This paper analyzes existing, publicly available data. These accession numbers for the datasets are listed in the key resources table.
(2) This paper does not report original code.
(3) Any additional information required to reanalyze the data reported in this paper is available from the lead contact upon request.

### Experimental model and study participant details

#### Preparation of PCa tissues

Some of the PCa tissues were collected after radical prostatectomy according to the pathological diagnosis of prostate biopsy. The freshly obtained PCa tissues were snap-frozen on dry ice and embedded in a freezing and embedding compound at Optimal Cutting Temperature (OCT). The OCT-embedded tissues were cryosectioned at a 10 μm thickness and placed on Visium Spatial slides. All procedures were implemented based on guidelines of the Ethics Committee of Beijing Tongren Hospital, affiliated with Capital Medical University.

#### Ethics approval and consent to participate

Human prostate samples were provided by Beijing Tongren Hospital. Ethical consent was approved by the Ethics Committee of Beijing Tongren Hospital, affiliated with Capital Medical University.

### Method details

#### RNA quality assessment

The quality of RNA in the tissue blocks was assessed by calculating the RNA integrity number (RIN) of freshly collected PCa tissue. Tissue sections with an RIN ≥7 were selected for placement on Visium Spatial slides.

#### Preparation of spatially barcoded arrays, staining and imaging

The Visium Spatial Gene Expression Slide & Reagent kit (10× Genomics, CA, USA) was used to generate spatially barcoded cDNA from tissue sections according to the manufacturer’s instructions. The Visium Spatial slide is a Codelink activated microscope glass slide upon which poly(dT)VN oligonucleotides (IDT) are distributed. The arrays on the slide were designed so that 4992 (64 × 78) spots containing unique barcoded oligonucleotides with poly(dT)VN were printed in each 6.5 × 6.5 mm^2^ capture area. The diameter of each spot was 55 μm, and the center-to-center distance of two adjacent spots was 100 μm (Figure 1). The fiducial frame (red spots) was printed as a border around the capture areas to maintain orientation. The Visium Spatial Tissue Optimization Slide with PCa tissue sections was subjected to methanol fixation and hematoxylin-eosin (H&E) staining.

#### Tissue optimization and permeabilization

In the quality control experiment, the permeabilization conditions for the tissue sections were optimized prior to spatial barcoding experiments to maximize mRNA yields in tissue sections. The experiment was performed by utilizing the Visium Spatial Tissue Optimization Slide & Reagent kit (10× Genomics) according to the manufacturer’s instructions. The optimal permeation time (with the strongest fluorescence signal intensity and the lowest dispersion among tested times of 30 min, 24 min 18 min, 12 min, 6 min, and 3 min) was determined by fluorescence imaging after fluorescence cDNA synthesis and tissue removal. The glass slides were scanned using an Olympus BX53 microscope (Olympus, Shinjuku, Tokyo, Japan), and signal intensities were investigated using the cellSens Dimension Software (Olympus) system. The cDNA fluorescence signals should be consistent with the structure of the tissue section shown by histology. This step was performed to ensure that all array sequences necessary for mRNA capture without a spatial barcode were present under the optimal permeabilization conditions. Then, a penetration operation was performed based on the optimal permeation time.

#### Reverse transcription, second-strand cDNA synthesis, and cDNA amplification

The polyadenylated mRNAs released from the overlying PCa cells were captured by primers (IDT) in the spots. Reverse transcription was performed to synthesize spatially barcoded, full-length cDNA (from polyadenylated mRNA on the slide) through the incubation of the permeabilized PCa sections with RT Master Mix reagents. Second-strand was synthesized by adding Second Strand Mix to the PCa sections on the slide. The cDNAs were denatured and transferred from each capture area to the corresponding tube and then amplified via Polymerase Chain Reaction (PCR) to generate a sufficient mass of DNA for library construction.

#### Library construction of cDNA and sequencing

Libraries of tissue sections were generated according to the 10× Genomics Visium library preparation protocol. cDNA amplicon size was optimized via enzymatic fragmentation and size selection. P5, P7, i5 and i7 sample indexes and TruSeq Read 2 sequences were added by performing end repair, tailing, adaptor ligation, and PCR. The final libraries containing the P5 and P7 primers were used for Illumina amplification. After library construction, 150PE-mode sequencing was carried out on the Illumina NovaSeq600 platform (Illumina, CA, USA).

#### Quality control of sequencing reads and genomic alignment

Primary quality control of sequencing reads was conducted according to the following criteria: (1) number of >3 for single-end sequencing reads (apart from those in paired reads); (2) quality value of a single-end sequencing read <5 and base ratio ≥20% (paired reads were removed); and (3) adapter sequences were removed (adapters should match ≥8 bp).

Space Ranger (10× Genomics) was used for genome alignment, in which the splicing-aware alignment of reads to the genome was performed by STAR aligner. After alignment, Space Ranger assesses each read as exonic, intronic, or intergenic and aligns the reads confidently to the genome based on the transcript annotation GTF file.

#### Analysis of gene expression by OmniAnalyzer Pro

OmniAnalyzer Pro (Analytical BioSciences Limited (Abiosciences), Beijing, China) was used in this study to analyze transcriptomes at spatial dimensions. The algorithm of this software was developed by Scanpy and Abiosciences. In spatial transcriptomic datasets, the spatial barcode information and reads of each spot are integrated via a processing algorithm to show the capture area of PCa tissue. The transcriptome profiling data from Space Ranger were subjected to quality control (cell filtering and gene filtering) and then transformed to transcripts per kilobase of exome per million (TPM) mapped reads.

#### Dimensionality reduction

To visually divide the spatial features of spots into different subgroups according to the expression patterns of whole genes, we performed principal component analysis (PCA) via OmniAnalyzer Pro. This pipeline uses the scipy.spare.linalg ARPACK implementation of the truncated singular value decomposition (SVD) to change the dimensionality of these datasets from (spots × genes) to (spots × M), where M is the PCA number of components (50 in filtered data and 15 in the inferCNV). To visualize data in two-dimensional space, OmniAnalyzer Pro applies the dimensional reduction method of UMAP. Additionally, Seurat cluster analysis was carried out for the spot matrix generated by OmniAnalyzer Pro, and Seurat passed the PCA-reduced data to UMAP. The spots were colored differently according to the different clusters. These spots were overlaid on the histological images to visualize the transcriptome-wide expression information and histological characteristics simultaneously.

#### Inferred copy number variation (inferCNV) analysis based on spatial transcriptomics

Genomic regions are commonly altered during PCa progression. Copy number analysis helps to predict clonal hierarchies and tumor malignancy.^4^^,^^5^^,^^6^^,^^7^^,^^8^^,^^25^^,^^26^ The monoclonal origin of multifocal PCa is derived from identical genomic copy number changes within the same case.^4^

To identify evidence of somatic large-scale chromosomal copy number alterations, CNVs were inferred based on transcriptomic data via the Python reimplementation of inferCNV. The data analysis was performed by calculating the transcription intensity of genes across positions of the PCa genome from each spot in comparison with a set of reference spots (in the cluster of GS (3 + 3)). The expression intensities of local genome fragments across each chromosome are illustrated in a heatmap. The average CNV score was evaluated to determine the malignant identities of PCa cells by averaging CNV modifications across 22 autosomes.^25^^,^^26^

#### Diffusion pseudotime (DPT)

DPT was performed to infer the progression of PCa through geodesic distance along the graph (https://scanpy.readthedocs.io/en/stable/generated/scanpy.tl.dpt.html). It can reconstruct the developmental progression of a biological process from snapshot data and identify transient or metastable states, branching decisions and differentiation endpoints.

#### Partition-based graph abstraction (PAGA)

PAGA preserves the global topology of single-cell RNA-sequence data and allows data to be analyzed at different resolutions. It was a further developed version of DPT that could discrete cell-to-cell variation based on continuous and disconnected structures and consistently predict gene expression changes and developmental trajectories using a transcriptome dataset. An interpretable abstracted graph-like map (PAGA graph) was generated to reconcile clusters with trajectory inference. In the PAGA graph of partitions, edge weights represent confidence in the presence of connections. A much simpler representation of the manifold data is obtained by thresholding this confidence in “paga()”, which is nonetheless faithful to the topology of the manifold. The specific method and trajectory-inference algorithms are described in https://scanpy.readthedocs.io/en/stable/generated/scanpy.tl.paga.html.

#### Analysis of differential expression and marker genes

Differential expression analysis facilitates the exploration of gene expression differences between clusters. The expression distribution of the input genes is shown in a dot plot or heatmap. Marker gene analysis was performed by OmniAnalyzer Pro to determine the top differentially expressed genes (DEGs) between two or more clusters. Wilcoxon-test was used to identify DEGs in the comparisons of one cluster versus (vs.) another or the rest. The results are shown in violin plots. Lower Q-values in Wilcoxon-test is associated with heterogeneity of gene expression in these comparisons.

#### Gene Ontology (GO) and Kyoto Encyclopedia of Genes and Genomes (KEGG) enrichment analysis

R software (4.0.3) was used for enrichment analysis. The DEGs associated with different biological functions and signaling pathways were assessed via GO function and KEGG pathway enrichment analyses by the R packages “clusterProfiler” and “enrichplot”.

#### Bioinformatic analysis using the prostate cancer of The Cancer Genome Atlas (TCGA-PRAD) database

The DEGs related to PCa progression were verified in the TCGA-PRAD dataset, which was downloaded from the Genomic Data Commons (GDC) Data Portal (https://portal.gdc.cancer.gov/). The expression of DEGs associated with various clinicopathological characteristics (such as GS and pathological T stage (pT)) was compared using TPM data. The R packages “limma”, “reshape2”, and “ggpubr” were used for these analyses. Survival analyses of recurrence-free survival (RFS) and progression-free survival (PFS) were conducted using Kaplan‒Meier curves and via Cox regression by the R packages “survival” and “survminer”. The RFS data was extracted from the follow-up phenotype file as we previously described,^62^^,^^63^ and the PFS data was obtained from the UCSC Xena website (https://xenabrowser.net/datapages/).^64^ Intersecting DEGs between the analyses of clinicopathological characteristics and survival were summarized with the R packages “VennDiagram”, “grid”, “futile.logger”, and “formatR”.

### Quantification and statistical analysis

OmniAnalyzer Pro, R software (4.0.3), and Microsoft Excel 2019 software (Microsoft Corp., Redmond, WA, USA) were used to conduct the statistical analyses. Wilcoxon-test with P- and Q-value calculations were used to analyze continuous data. Survival analysis was performed by utilizing Kapan‒Meier curves with a log rank test and univariate Cox regression models with hazard ratios (HRs).
