# Supplementary material for: Visium spatial transcriptomics reveals intratumor heterogeneity and profiles of Gleason score progression in prostate cancer
Source: iScience. 2023 Nov 10;26(12):108429. doi: 10.1016/j.isci.2023.108429 (PMC10698261; doi:10.1016/j.isci.2023.108429)
Supplement: Document S1. Figures S1–S11 and Tables S1–S14 [file mmc1.pdf]

iScience, Volume *26*

## Supplemental information

# Visium spatial transcriptomics reveals intratumor heterogeneity and profiles of Gleason score progression in prostate cancer

**Yongjun Quan, Hong Zhang, Mingdong Wang, and Hao Ping**

## SUPPLEMENTAL FIGURES

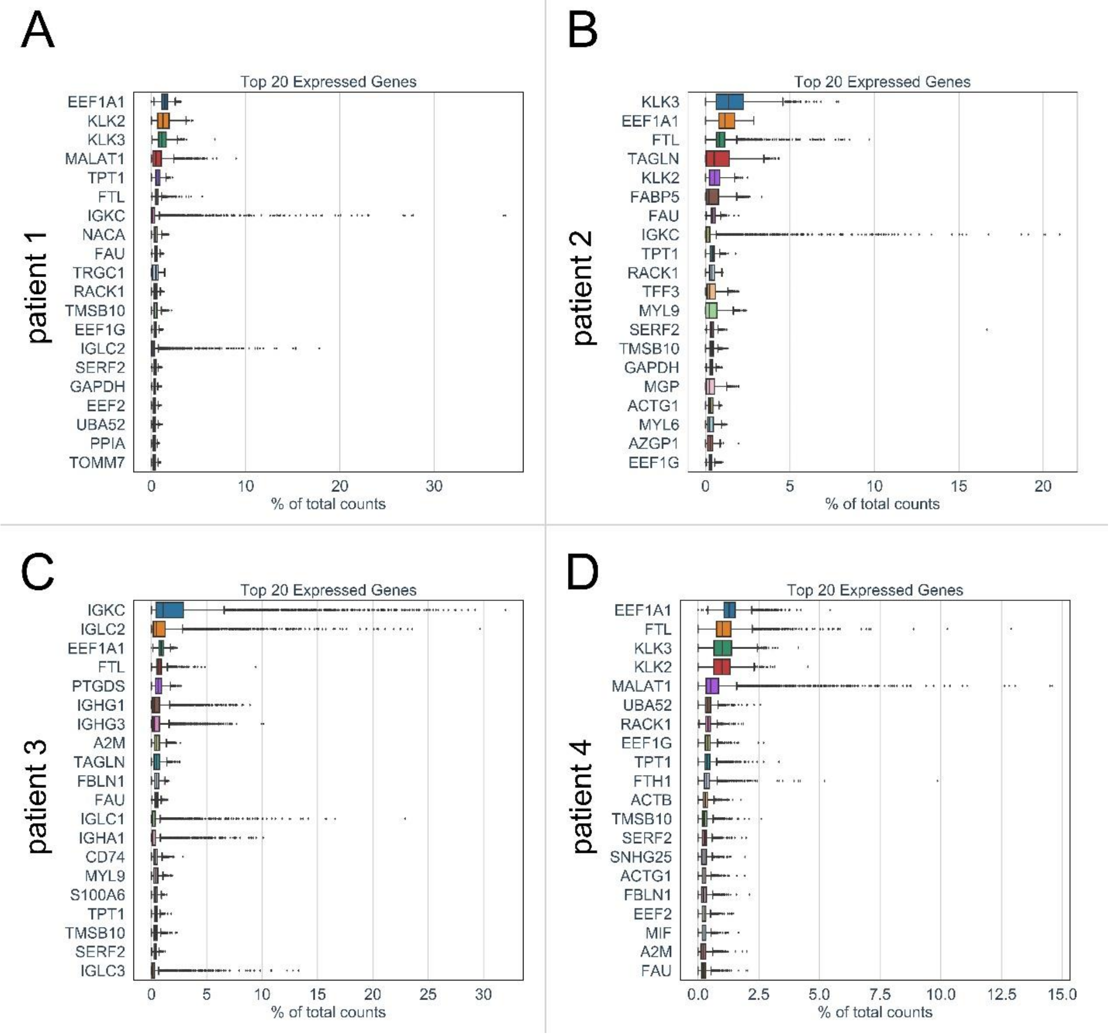


**Figure S1. The top expressed genes in patients 1-4. Related to Figure 1, 2, 5.**

(A-D) The top 20 expressed genes in patient 1 (A), patient 2 (B), patient 3 (C), and patient 4 (D) according to ST analysis.

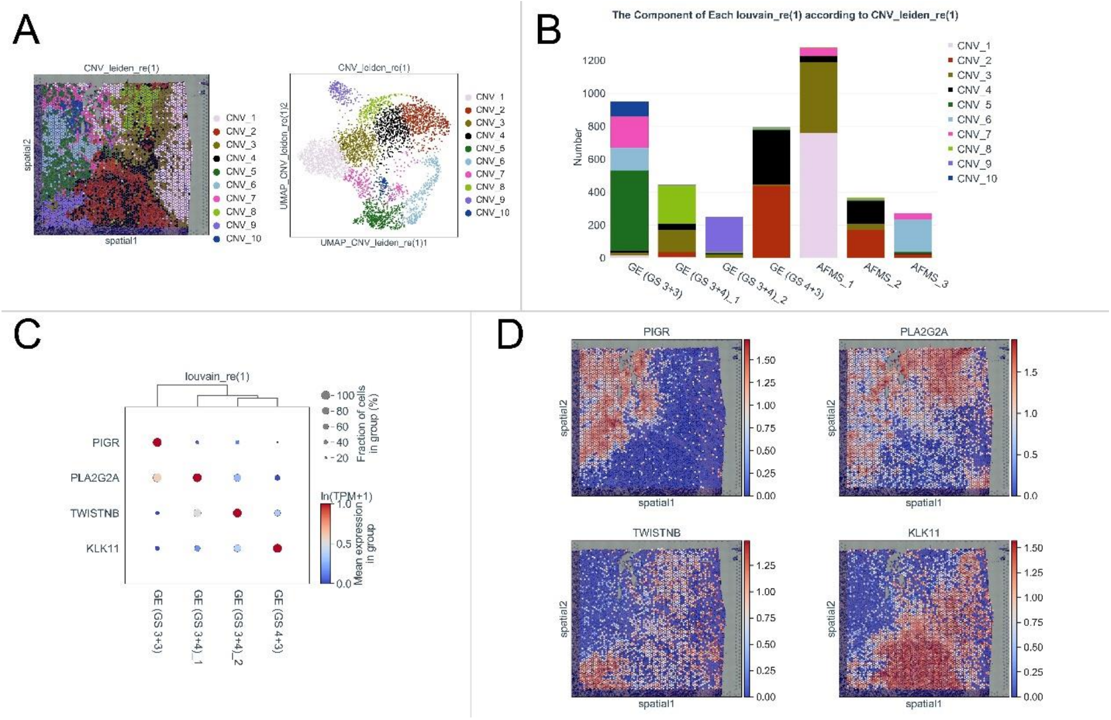


**Figure S2. Genomic heterogeneity and crucial factors in GE subgroups of patient 1. Related to Figure 2.**

(A) The classifications across all the spots on the basis of the CNV values were determined via PCA. The spots of the subclusters overlaid on the histological image were annotated with CNV_1 to CNV_10 (left) and visualized by UMAP (right). (B) The components of histological subgroups according to CNV clusters are shown in histograms. The x-axis represents histological subgroups, and the y-axis represents the number of spots in CNV clusters. (C-D) The expression levels of the crucial factors that are generally expressed in the different GSs are shown in a bubble plot (C) and spatial activity maps in the histological image (D).

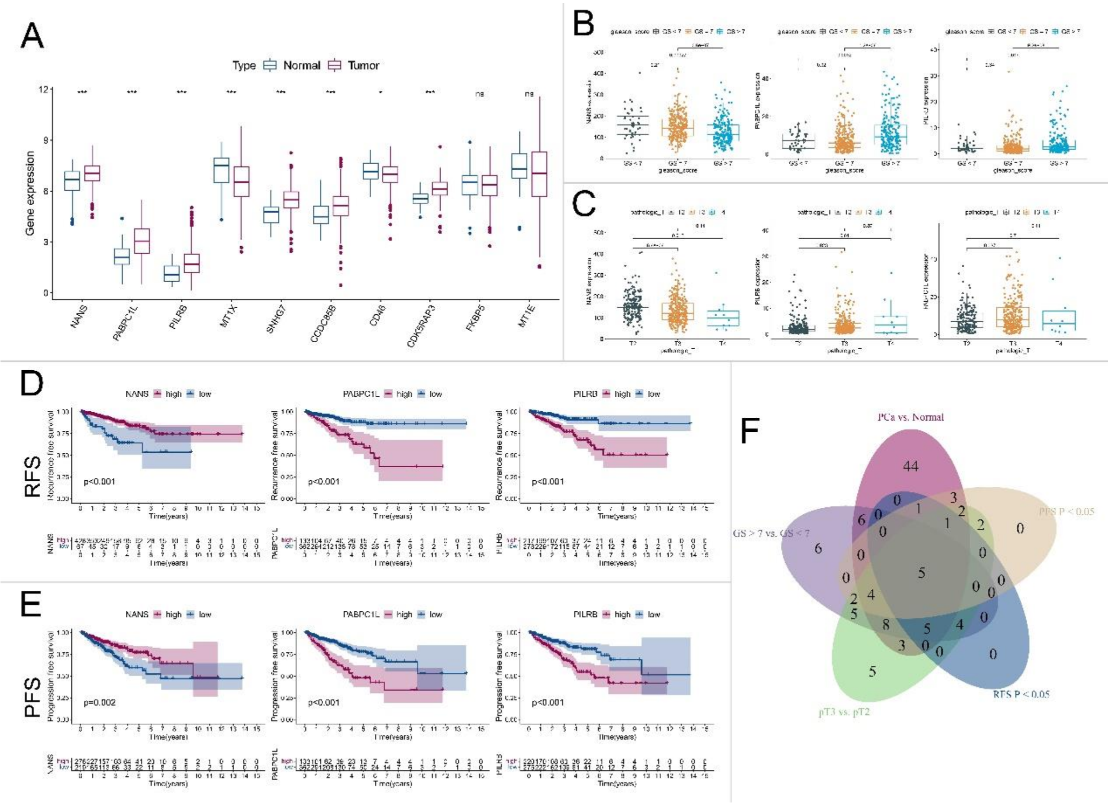


**Figure S3. Verification of GS development-related genes using the TCGA-PRAD dataset in patient 1. Related to Figure 3, 4.**

(A) The genes with coincident trends in the TCGA-PRAD dataset and ST analysis are summarized in Table S7, and the expression levels of the 10 genes in PCa and normal prostate tissues of TCGA-PRAD are shown in a boxplot. The median ± interquartile ranges are shown in the graph. ns $P > 0.05$; * $P < 0.05$; ***$P < 0.001$. (B-C) Distribution of the 3 representative genes (NANS, PABPC1L, and PILRB) in the comparisons of TCGA-PRAD data stratified by GS (GS > 7 vs. GS < 7) (B) and pT (T3 vs. T2) (C). Median ± interquartile range values are shown in the boxplot, and P-values in Wilcoxon-test are presented above each pair of comparisons. (D-E) The 495 TCGA-PRAD patients were divided into two groups according to the optimum cutoff of the expression value of each gene with the lowest log-rank P-value in the survival analysis. The prognostic outcomes of RFS (D) and PFS (E) in the 3 genes among the two groups are shown in Kaplan–Meier curves. The log-rank P-values are shown in the graph, and the numbers at risk are shown at the bottom. (F) The expression levels of GS-associated genes were detected in association with various clinicopathological characteristics and prognoses of the TCGA-PRAD dataset. The simultaneously dysregulated genes in the comparisons of PCa vs. normal, GS > 7 vs. GS < 7, T3 vs. T2 ($P < 0.05$), and RFS and PFS (P-value of univariate Cox regression analysis < 0.05) are shown in a Venn diagram.

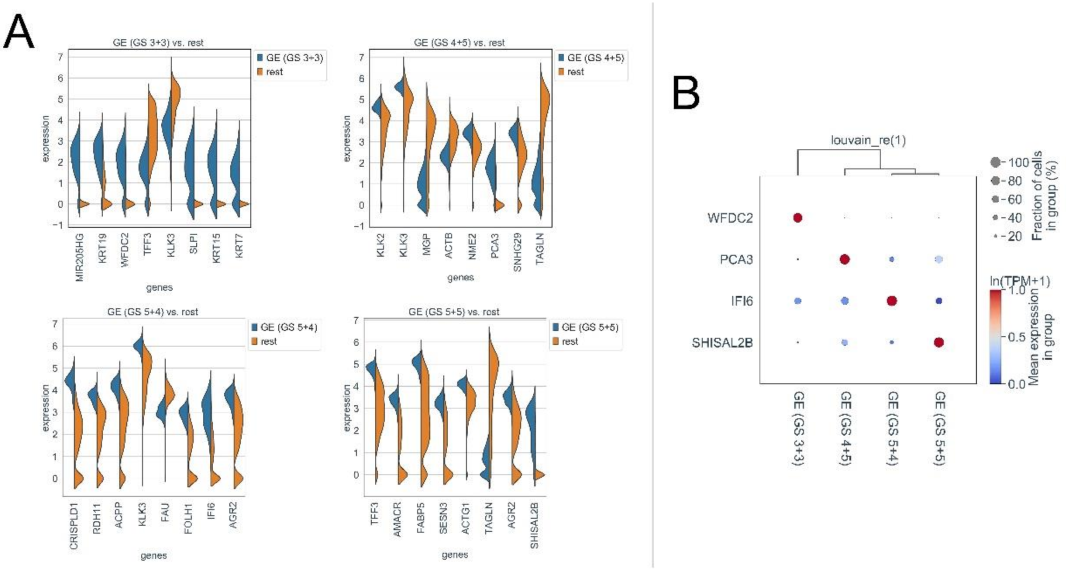


**Figure S4. Crucial factors that are generally expressed in the GE subgroups of patient 2. Related to Figure 5.**

(A) DEGs in paired comparisons between each individual GS and the others are shown in violin plots. The x-axis represents 8 genes with the lowest Q-values in Wilcoxon-test, and the y-axis represents the expression levels of each individual GE subcluster (left half) and the others (right half) in the violin plot. (B) The expression levels of the crucial factors that are generally expressed in the different GSs are shown in a bubble plot.

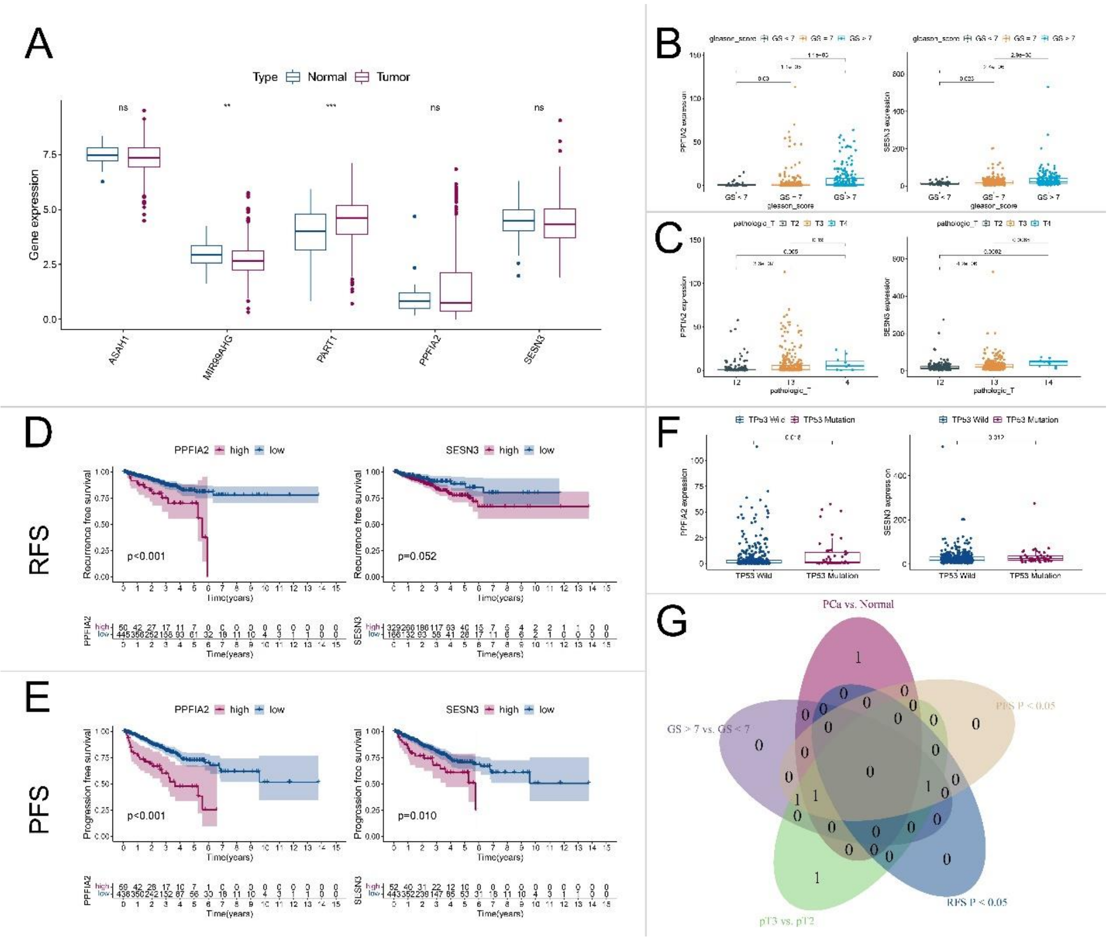


**Figure S5. Verification of GS development-related genes using the TCGA-PRAD dataset in patient 2. Related to Figure 6.**

(A) The 5 simultaneously upregulated genes in the four GS comparisons are summarized in Figure 6 and Table S14. The expression levels of the 5 genes in PCa and normal prostate tissues of TCGA-PRAD are shown in a boxplot. The median ± interquartile ranges are shown in the graph. ns $P > 0.05$; ** $P < 0.01$; ***$P < 0.001$. (B-C) The 2 genes (PPFIA2 and SESN3) showing consistent trends in the TCGA-PRAD dataset and ST analysis were compared in TCGA-PRAD data stratified by GS (GS > 7 vs. GS < 7) (B) and pT (T3 vs. T2) (C). Median ± interquartile range values are shown in the boxplot, and P-values are presented above each pair of comparisons. (D-E) The 495 TCGA-PRAD patients were divided into two groups according to the expression of each gene as described in Figures S3D-S3E. The prognostic outcomes of RFS (D) and PFS (E) in the 2 genes between the two groups are shown in Kaplan–Meier curves. The log-rank P-values are shown in the graph, and the numbers at risk are shown at the bottom. (F) The expression levels of the 2 genes were compared according to TCGA-PRAD data stratified by TP53 mutation status (TP53 mutation vs. wild type). Median ± interquartile range values are shown in the boxplots, and P-values are presented above

each pair of comparisons. (G) The expression levels of 5 GS-associated genes were evaluated in association with various clinicopathological characteristics and prognoses of the TCGA-PRAD dataset. The simultaneously dysregulated genes in the comparisons of PCa vs. normal, GS > 7 vs. GS < 7, T3 vs. T2 (P < 0.05), and RFS and PFS (P-value of univariate Cox regression analysis < 0.05) are shown in a Venn diagram.

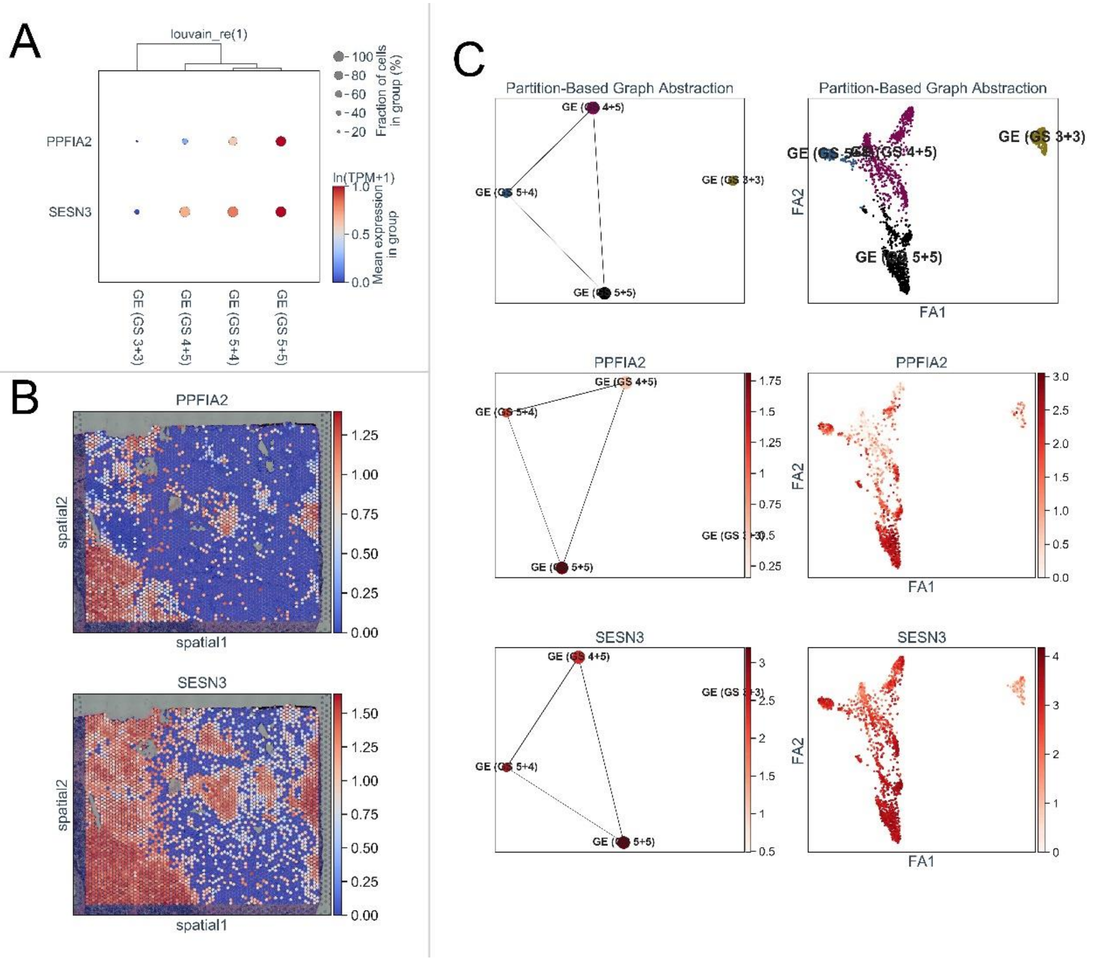


**Figure S6. Expression patterns of simultaneously altered genes in the TCGA-PRAD dataset and ST analysis in patient 2. Related to Figure 6.**

(A) The expression patterns of the 2 genes (PPFIA2 and SESN3) showing consistent trends in the TCGA-PRAD dataset and ST analysis are shown in a bubble plot. The x-axis represents four GE subclusters, and the y-axis represents 2 genes. Red: high expression, blue: low expression. (B) The expression levels of the 2 genes are shown in spatial activity maps in the histological image. (C) The expression and distribution of the 2 genes on the basis of retaining the GE subclusters (left) and each spot (right) map in PAGA plots of four GE subclusters.

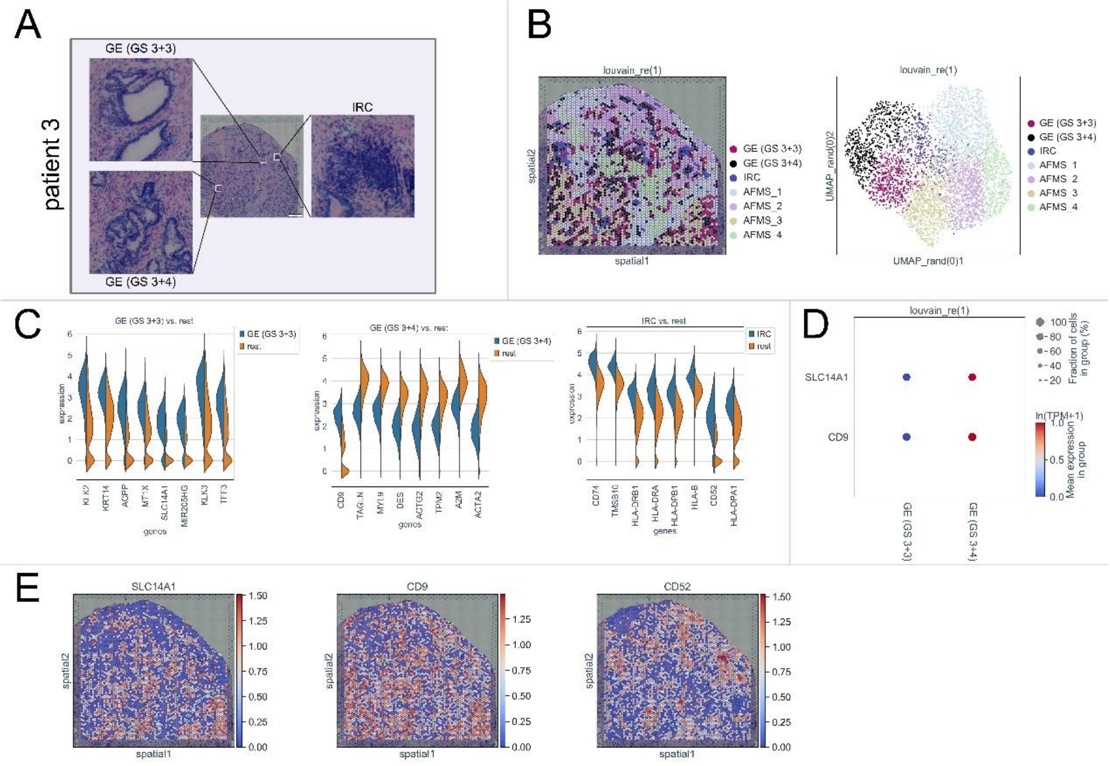


**Figure S7. Transcriptomic heterogeneity of PCa tissue from patient 3. Related to Figure 1, 7.**

(A) H&E-stained image of PCa from patient 3. Three different histological structures of lesions are enlarged and annotated with GSs or IRC. (B) The genetic classification across all spots was analyzed via PCA. The spots of the subclusters overlaid on the histological image were annotated with GSs, IRC, and AFMS on the basis of histological structures (left) and visualized by UMAP (right). (C) DEGs in paired comparisons between each individual GS and the others are shown in violin plots. The x-axis represents the 8 genes with the lowest Q-values in Wilcoxon-test, and the y-axis represents the expression levels of each GE (left half) and the others (right half) in the violin plot. (D-E) The expression levels of the crucial factors that are generally expressed in the different GSs are shown in a bubble plot (D) and spatial activity maps in the histological image (E).

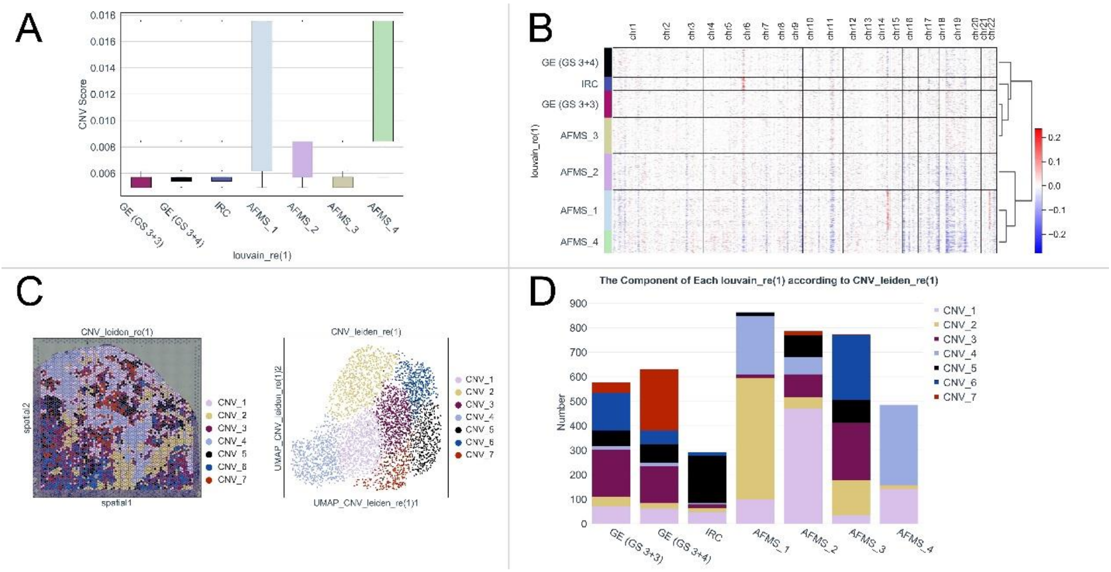


**Figure S8. Genomic heterogeneity of PCa tissue from patient 3. Related to Figure 1, 7.**

(A) The CNV value of each gene was calculated by inferCNV, and the average CNV score of each subgroup is shown in a histogram. (B) The chromosomal landscape of inferCNVs distinguishing subgroups of GEs, IRC, and AFMS is shown in a heatmap. The horizontal axis represents chr1 to chr22, the left vertical axis represents the spot subclusters, and the right vertical axis represents unsupervised hierarchical clustering. Red: gain of copies, blue: loss of copies. (C) The classifications across all the spots on the basis of the CNV values were determined via PCA. The spots of the subclusters overlaid on the histological image were annotated with CNV_1 to CNV_7 (left) and visualized by UMAP (right). (D) The components of histological subgroups according to CNV clusters are shown in histograms. The x-axis represents histological subgroups, and the y-axis represents the number of spots in CNV clusters.

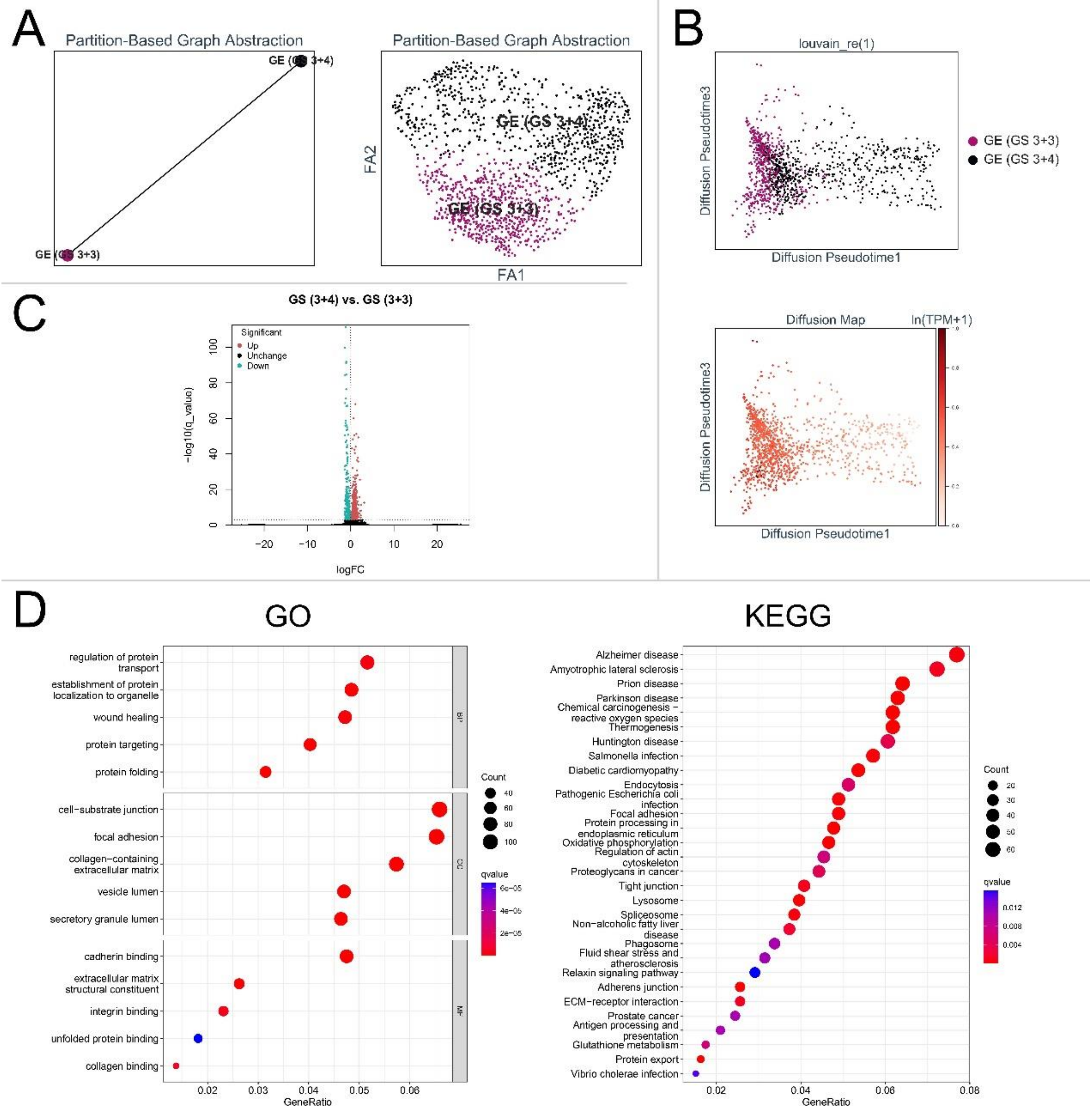


**Figure S9. Spatial gene expression patterns during GS progression in patient 3. Related to Figure 1, 7.**

(A) The inference of the GS development trajectory on the basis of retaining the GE subclusters (left) and each spot (right) map was performed via a static PAGA plot. (B) The developmental paths of different GSs (top) were inferred through DPT analysis. The diffusion map (down) was used to construct appropriate coordinates. (C) The DEGs in the comparisons among GE clusters of GS (3+4) vs. GS (3+3) were analyzed, and the significantly altered genes (Q-value < 0.001) were visualized in a Volcano plot. Red spots: high expression, green spots: low expression, black spots: not significant between the comparison. (D) The GO (left) and KEGG (right) enrichment analyses using the DEGs in the comparison between GS (3+4) vs. GS (3+3) are shown in dot plots.

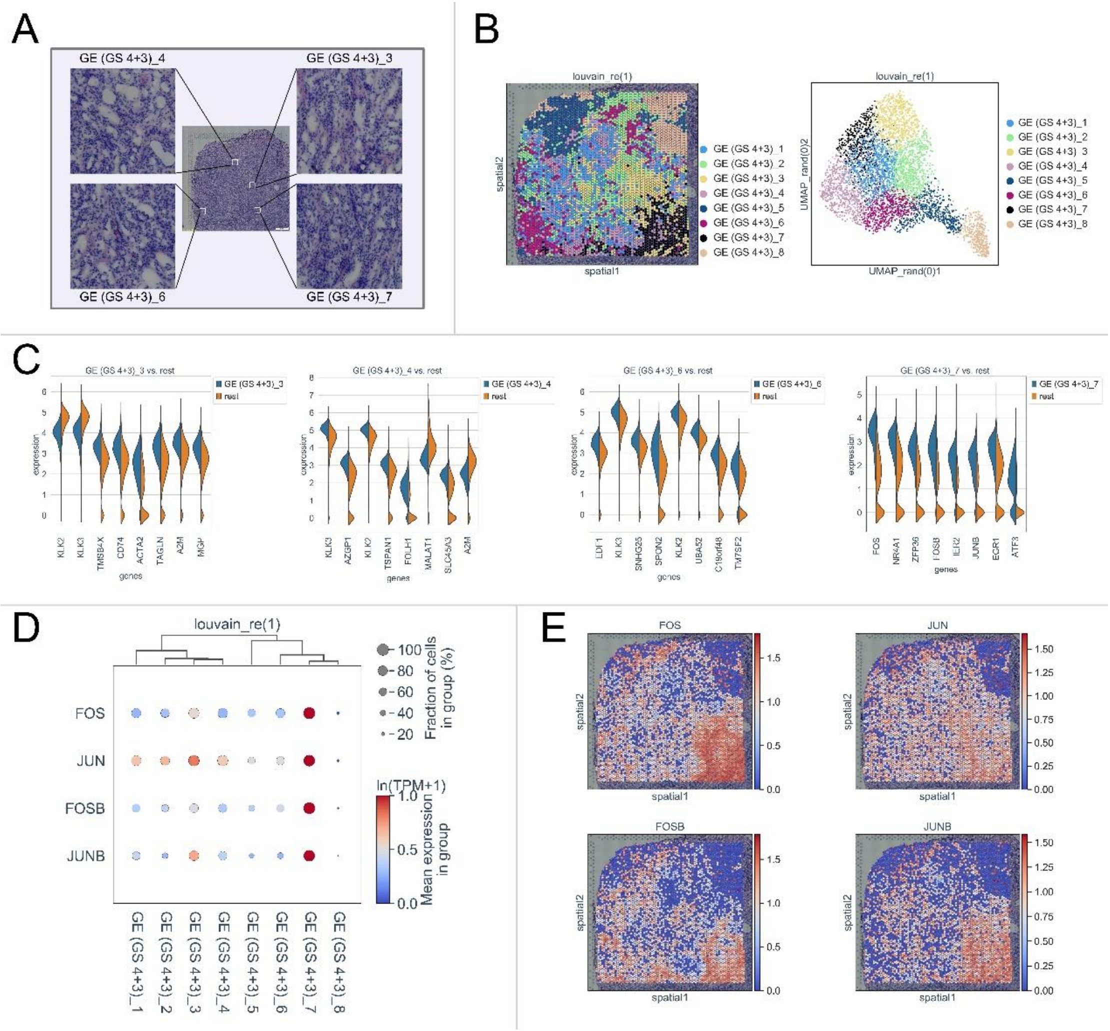

**Figure S10. Transcriptomic and genomic heterogeneity of PCa tissue from patient 4. Related to Figure 1.**

(A) H&E-stained image of PCa from patient 4. Four differentially classified histological structures of lesions are enlarged and annotated with GSs. (B) The genetic classifications across all spots were analyzed via PCA. The spots of the subclusters overlaid on the histological images were annotated with GSs on the basis of histological structures (left) and visualized by UMAP (right). (C) DEGs in paired comparisons between four selected GSs (GS (4+3)_3, GS (4+3)_4, GS (4+3)_6, and GS (4+3)_7) and the others are respectively shown in violin plots. The x-axis represents the 8 genes with the lowest Q-values in Wilcoxon-test, and the y-axis represents the expression levels of each GE (left half) and the others (right half) in the violin plot. (D-E) The expression levels of the immediate early response genes that are generally expressed in GS (4+3)_7 are shown in a bubble plot (D) and spatial activity maps in the histological image (E).

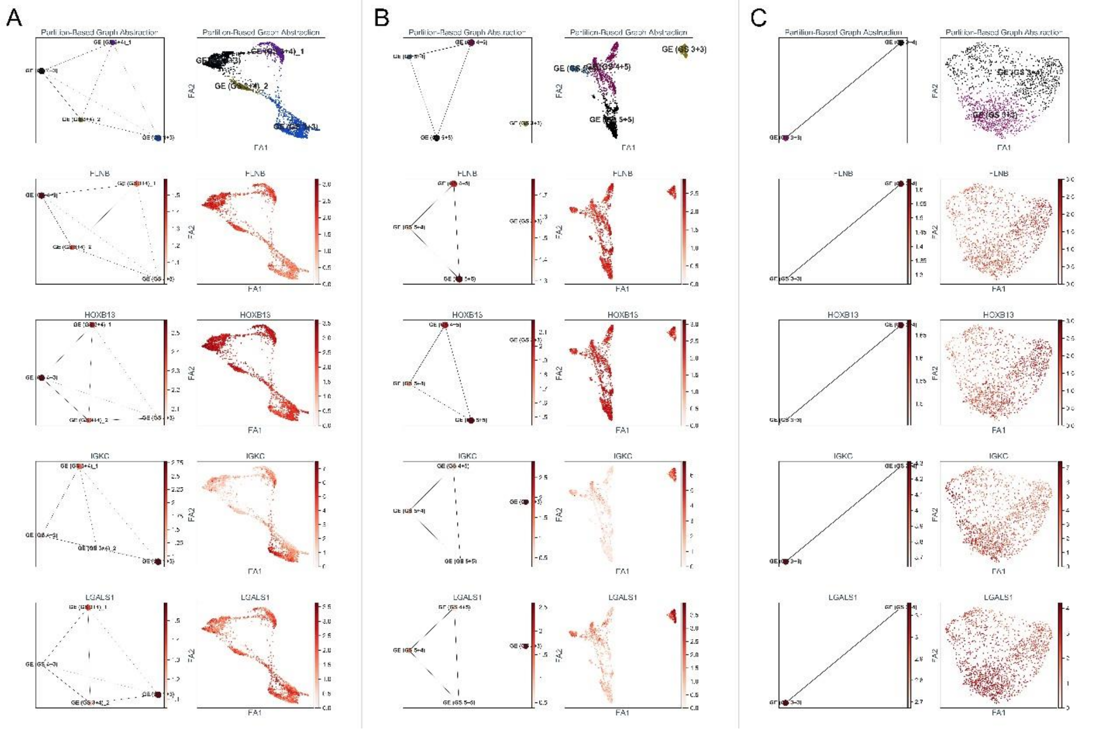


**Figure S11. Simultaneously dysregulated genes during GS development in patients 1-3. Related to Figure 7.**

(A-C) The expression and distribution of the 4 genes mentioned in Figure 7C in PAGA plots of GE subclusters in patients 1-3 (A-C).

# SUPPLEMENTARY TABLES

**Supplementary Table 1. The expression levels of GS development related genes from patient 1 in TCGA-PRAD and normal tissues. Related to Figure 3, 4.**

| Gene | Normal (median) | Tumor (median) | pValue | P symbol[a] |
|---|---|---|---|---|
| ABCC4 | 6.593930438 | 7.908853729 | 1.03068E-13 | *** |
| ACSM1 | 2.86829741 | 5.973178448 | 3.90445E-14 | *** |
| ADIRF | 0 | 0 | 0.096571769 | ns |
| AGR2 | 8.217254548 | 8.985781041 | 0.015951803 | * |
| ANXA5 | 8.583533948 | 8.636357443 | 0.022388971 | * |
| APLP2 | 8.929596578 | 8.955953815 | 0.034483623 | * |
| ARHGDIB | 6.435740891 | 6.560222765 | 0.584762666 | ns |
| ARPC1B | 5.717574752 | 5.706897672 | 0.566035066 | ns |
| ASAH1 | 7.465753583 | 7.364302777 | 0.089008799 | ns |
| B4GALT1 | 6.945082418 | 6.61543469 | 0.000110095 | *** |
| CACYBP | 5.172675086 | 5.002125957 | 0.041877192 | * |
| CAPN2 | 5.860133145 | 5.733645291 | 0.11185805 | ns |
| CD46 | 7.155240975 | 6.974884895 | 0.020422966 | * |
| CD59 | 7.772605534 | 7.257102104 | 2.57129E-15 | *** |
| CDK2AP1 | 6.434706914 | 6.263163078 | 0.169193901 | ns |
| CHMP5 | 6.982096522 | 6.878686111 | 0.214883786 | ns |
| CLTC | 6.993522514 | 7.054432839 | 0.523491621 | ns |
| CNIH4 | 4.199268544 | 4.058861084 | 0.010299176 | * |
| COPB1 | 6.907403152 | 6.938212855 | 0.281336355 | ns |
| COPB2 | 6.433036727 | 6.675127156 | 0.008969468 | ** |
| CRIP2 | 5.67973353 | 5.372861927 | 0.008732089 | ** |
| DEGS1 | 7.607314391 | 7.859758395 | 0.001530644 | ** |
| DNAJA1 | 7.830365273 | 7.783130518 | 0.826124376 | ns |
| DSG2 | 6.335029248 | 6.075336451 | 0.282154709 | ns |
| EFR3A | 5.711924292 | 5.448925392 | 0.007710903 | ** |
| EIF4G2 | 8.466894706 | 8.147299066 | 9.18661E-05 | *** |
| EPCAM | 7.289068685 | 8.470716621 | 1.12658E-18 | *** |
| F5 | 2.911523716 | 3.916529192 | 2.02884E-05 | *** |
| FKBP5 | 6.508461036 | 6.354881781 | 0.670384718 | ns |
| GFPT1 | 6.195238388 | 6.53104578 | 0.003102726 | ** |
| GPR160 | 5.033322885 | 6.04417595 | 1.67034E-13 | *** |
| HEXB | 6.569498916 | 6.540508418 | 0.227670553 | ns |
| HNRNPC | 7.323372599 | 7.473089581 | 0.006854503 | ** |

| | | | | |
|---|---|---|---|---|
| HSP90AA1 | 9.45891362 | 9.213572564 | 0.00818513 | ** |
| HSPA5 | 9.183914901 | 9.411891208 | 0.012754057 | * |
| HSPA8 | 9.292439808 | 9.536532229 | 0.012365182 | * |
| HTATIP2 | 5.070108069 | 4.758691731 | 0.007401839 | ** |
| ISG15 | 5.713059692 | 6.25100912 | 7.7083E-06 | *** |
| LAPTM4A | 9.331545483 | 8.910209482 | 1.24595E-15 | *** |
| LMAN1 | 6.718117512 | 6.752419452 | 0.948913026 | ns |
| LRP10 | 7.051093653 | 6.786091853 | 0.004219539 | ** |
| MANF | 6.582176047 | 7.161030072 | 6.44651E-12 | *** |
| MANSC1 | 5.039983388 | 5.087395797 | 0.752862398 | ns |
| MORF4L1 | 6.771808244 | 6.508822868 | 0.000130725 | *** |
| MPC2 | 7.394843705 | 8.075080114 | 1.89453E-10 | *** |
| MPZL1 | 5.92202225 | 6.046866222 | 0.018336163 | * |
| MT1E | 7.304224639 | 7.053581657 | 0.060225999 | ns |
| MT1X | 7.513273926 | 6.533308394 | 8.13397E-05 | *** |
| MYL12A | 7.519007749 | 7.40777931 | 0.447976023 | ns |
| MYL12B | 9.178616221 | 9.053684281 | 0.089353291 | ns |
| NANS | 6.666522101 | 7.060626767 | 2.01229E-05 | *** |
| PDIA4 | 7.973685491 | 8.544086188 | 9.63255E-08 | *** |
| PDXDC1 | 6.386654195 | 6.660701909 | 3.99238E-05 | *** |
| PIGR | 5.998792995 | 5.34465525 | 0.105407063 | ns |
| PPP1R1B | 6.85127065 | 7.229676358 | 0.323786642 | ns |
| PSMA6 | 3.399223553 | 3.457875423 | 0.403850311 | ns |
| PSME1 | 8.330073027 | 8.383293455 | 0.082826539 | ns |
| RAB1A | 7.589583998 | 7.526315345 | 0.54699091 | ns |
| RPN1 | 8.230381328 | 8.335233124 | 0.022227988 | * |
| S100A10 | 7.752501939 | 7.504039543 | 0.4673744 | ns |
| SAT1 | 8.866423696 | 9.297755432 | 3.26433E-06 | *** |
| SEC61B | 7.942848788 | 8.327292599 | 2.11283E-09 | *** |
| SH3YL1 | 4.294075713 | 4.345776019 | 0.257918078 | ns |
| SNRPB2 | 5.519676068 | 5.611039261 | 0.193062185 | ns |
| SPINT2 | 8.065579868 | 8.272985225 | 0.001483136 | ** |
| SPOCK1 | 7.141467247 | 7.706806799 | 5.61845E-05 | *** |
| SRP9 | 8.034119566 | 7.945046968 | 0.393112703 | ns |
| SURF4 | 8.261129393 | 8.414743154 | 0.003360722 | ** |
| TIMM17A | 5.258658859 | 5.329905383 | 0.012111802 | * |
| TM9SF1 | 3.228019606 | 3.412670169 | 0.008755571 | ** |
| TMEM63A | 5.014697447 | 5.007408122 | 0.645227758 | ns |
| TNFSF10 | 7.382800661 | 7.557588532 | 0.021594079 | * |
| TOMM5 | 3.825056277 | 3.984025103 | 0.00013219 | *** |
| UAP1 | 6.369129009 | 7.279586439 | 2.30465E-13 | *** |
| YWHAQ | 8.617886595 | 8.650611279 | 0.124564508 | ns |
| ALKBH7 | 6.898611517 | 7.147601802 | 3.86772E-05 | *** |
| AZGP1 | 10.61472634 | 10.32120769 | 0.148894601 | ns |

| | | | | |
|---|---|---|---|---|
| CBFA2T2 | 4.661663204 | 4.994351626 | 0.001138187 | ** |
| CCDC85B | 4.489811471 | 5.156405516 | 9.8682E-06 | *** |
| CD320 | 6.25122141 | 6.762864351 | 2.74673E-08 | *** |
| CDK5RAP3 | 5.561853226 | 6.132854415 | 1.33098E-13 | *** |
| CIRBP | 7.635584682 | 7.928485805 | 9.11229E-08 | *** |
| DANCR | 5.808266793 | 6.783723795 | 8.45557E-16 | *** |
| EEF2 | 11.26849216 | 11.82845348 | 2.96752E-12 | *** |
| EIF3B | 5.902275997 | 6.484334248 | 3.22027E-19 | *** |
| ERP29 | 7.905702308 | 8.176250457 | 8.13397E-05 | *** |
| FAM189A2 | 4.832773471 | 5.397708513 | 5.88793E-07 | *** |
| FAU | 9.299753537 | 9.366482213 | 0.013323483 | * |
| FLNB | 7.240807886 | 7.370792009 | 0.250295543 | ns |
| HSD17B6 | 5.867736118 | 5.311010411 | 0.075329789 | ns |
| IGFBP2 | 7.012920038 | 7.486213976 | 6.29164E-07 | *** |
| KLK11 | 7.154849668 | 6.19496699 | 0.0660703 | ns |
| KLK2 | 10.89507669 | 11.62299393 | 1.48181E-07 | *** |
| MAP2K2 | 6.173645115 | 6.502744782 | 5.64746E-09 | *** |
| MICOS13 | 6.111144288 | 6.379988243 | 2.64702E-06 | *** |
| MZT2B | 6.720859455 | 7.220180956 | 1.95645E-11 | *** |
| NAA10 | 4.138583257 | 4.588839939 | 1.6205E-14 | *** |
| NACA | 7.8305944 | 8.101682639 | 1.2194E-09 | *** |
| NDUFA11 | 5.120481189 | 5.169020228 | 0.034955047 | * |
| NDUFB11 | 7.459008046 | 7.79560982 | 1.18773E-08 | *** |
| NISCH | 6.0512261 | 5.910228734 | 0.188407992 | ns |
| NUMA1 | 6.55942559 | 6.922283494 | 6.63721E-05 | *** |
| PABPC1L | 2.077179632 | 3.049250006 | 4.17218E-09 | *** |
| PEBP1 | 9.706170323 | 9.822365923 | 0.009977642 | ** |
| PILRB | 1.082486422 | 1.691536398 | 6.41794E-08 | *** |
| PIN1 | 5.453641246 | 5.595835115 | 0.004884037 | ** |
| PLEKHH1 | 4.255863275 | 4.985370181 | 8.97519E-08 | *** |
| POLD4 | 4.664558702 | 5.132344874 | 1.73426E-09 | *** |
| RACK1 | 9.329070088 | 9.777879937 | 3.1466E-12 | *** |
| S100A13 | 4.899439163 | 4.485768139 | 1.20818E-05 | *** |
| SMS | 8.036645778 | 8.641219059 | 1.96612E-06 | *** |
| SND1 | 7.261856351 | 7.81476125 | 7.39494E-13 | *** |
| SNHG7 | 4.766691251 | 5.477454237 | 6.84716E-13 | *** |
| SORD | 7.722406071 | 7.759754391 | 0.831832137 | ns |
| TIMM13 | 6.339093689 | 6.988701726 | 9.56476E-19 | *** |
| TM7SF2 | 6.512344984 | 7.197501763 | 3.53337E-09 | *** |
| VSTM2L | 5.218252075 | 6.932867878 | 1.23866E-12 | *** |

[a] ns P > 0.05; * P < 0.05; ** P < 0.01; ***P < 0.001.

**Supplementary Table 2. Expression levels of GS development related genes from patient 1 in TCGA-PRAD data stratified by GS. Related to Figure 3, 4.**

| Gene | GS < 7 (median) | GS = 7 (median) | GS > 7 (median) | P (GS < 7 vs. GS > 7) | P symbol[a] |
|---|---|---|---|---|---|
| ABCC4 | 274.5179 | 279.00175 | 199.2805 | 0.140859 | ns |
| ACSM1 | 48.85425 | 72.8086 | 53.41567 | 0.757552 | ns |
| ADIRF | 0 | 0 | 0 | 0.329463 | ns |
| AGR2 | 458.9273 | 545.25325 | 433.557 | 0.481254 | ns |
| ANXA5 | 360.0067 | 403.7743 | 405.6947 | 0.016764 | * |
| APLP2 | 474.9407 | 519.4205 | 477.9448 | 0.504324 | ns |
| ARHGDIB | 86.61272 | 90.374555 | 100.2952 | 0.173654 | ns |
| ARPC1B | 45.31363 | 50.266275 | 55.21468 | 0.002134 | ** |
| ASAH1 | 155.1954 | 170.99065 | 155.681 | 0.561369 | ns |
| B4GALT1 | 94.51148 | 102.8561 | 93.54956 | 0.796105 | ns |
| CACYBP | 29.99609 | 30.41707 | 32.22811 | 0.001886 | ** |
| CAPN2 | 44.26105 | 48.801585 | 55.4135 | 0.001195 | ** |
| CD46 | 134.5124 | 128.63125 | 117.5166 | 0.180253 | ns |
| CD59 | 149.1216 | 160.61565 | 143.6523 | 0.259608 | ns |
| CDK2AP1 | 64.65061 | 76.013145 | 82.67009 | 3.63E-05 | *** |
| CHMP5 | 112.1068 | 117.5628 | 115.7305 | 0.130087 | ns |
| CLTC | 107.429 | 132.3793 | 137.5435 | 0.019205 | * |
| CNIH4 | 14.3391 | 15.25239 | 16.80307 | 1.8E-06 | *** |
| COPB1 | 102.9526 | 124.336 | 122.35 | 0.033645 | * |
| COPB2 | 88.04217 | 102.23385 | 102.3146 | 0.047166 | * |
| CRIP2 | 33.47403 | 33.657405 | 53.86416 | 1.17E-05 | *** |
| DEGS1 | 184.5161 | 216.58765 | 262.988 | 0.000163 | *** |
| DNAJA1 | 196.0199 | 219.31525 | 233.2561 | 0.000635 | *** |
| DSG2 | 53.98595 | 68.841675 | 66.91226 | 0.25672 | ns |
| EFR3A | 42.36005 | 45.38123 | 40.1085 | 0.757552 | ns |
| EIF4G2 | 254.2393 | 282.1447 | 289.1287 | 0.005351 | ** |
| EPCAM | 315.3095 | 362.4456 | 351.2502 | 0.311067 | ns |
| F5 | 7.798102 | 12.40687 | 17.38952 | 0.003423 | ** |
| FKBP5 | 96.74477 | 86.74441 | 71.93551 | 0.03178 | * |
| GFPT1 | 79.53935 | 98.129015 | 88.04565 | 0.657325 | ns |
| GPR160 | 52.69613 | 62.600925 | 71.70447 | 0.001602 | ** |
| HEXB | 90.22548 | 105.76465 | 85.0927 | 0.134197 | ns |
| HNRNPC | 150.707 | 173.8564 | 184.5116 | 0.00038 | *** |
| HSP90AA1 | 512.8392 | 601.45825 | 593.8896 | 0.01751 | * |
| HSPA5 | 557.1647 | 747.49405 | 637.469 | 0.419559 | ns |
| HSPA8 | 645.8609 | 785.42725 | 736.4847 | 0.026241 | * |

| | | | | | |
|---|---|---|---|---|---|
| HTATIP2 | 28.50323 | 26.92089 | 24.77133 | 0.485535 | ns |
| ISG15 | 69.87182 | 70.28685 | 81.00435 | 0.174378 | ns |
| LAPTM4A | 464.6716 | 479.9879 | 486.0199 | 0.149701 | ns |
| LMAN1 | 95.50543 | 112.5753 | 101.8705 | 0.707653 | ns |
| LRP10 | 100.6332 | 113.74075 | 108.1044 | 0.403928 | ns |
| MANF | 126.6432 | 144.3734 | 139.992 | 0.167941 | ns |
| MANSC1 | 32.72202 | 36.54419 | 30.25914 | 0.426175 | ns |
| MORF4L1 | 84.5455 | 87.800715 | 95.20988 | 0.003821 | ** |
| MPC2 | 276.6873 | 268.44375 | 269.2209 | 0.754075 | ns |
| MPZL1 | 61.06478 | 66.20879 | 62.55992 | 0.308888 | ns |
| MT1E | 200.1538 | 182.3095 | 86.63595 | 3.63E-05 | *** |
| MT1X | 120.44784 | 105.3406 | 71.92163 | 0.002183 | ** |
| MYL12A | 165.5639 | 167.2691 | 170.1224 | 0.432852 | ns |
| MYL12B | 516.6895 | 530.22545 | 541.5466 | 0.027824 | * |
| NANS | 157.3798 | 142.79225 | 111.928 | 0.000272 | *** |
| PDIA4 | 325.6689 | 417.3987 | 332.2999 | 0.941669 | ns |
| PDXDC1 | 91.42065 | 101.2604 | 99.76869 | 0.117266 | ns |
| PIGR | 25.35016 | 43.96375 | 31.78354 | 0.994527 | ns |
| PPP1R1B | 153.6621 | 176.30205 | 110.6715 | 0.029149 | * |
| PSMA6 | 9.213002 | 9.6106745 | 10.86608 | 0.000178 | *** |
| PSME1 | 313.4356 | 328.6185 | 338.8833 | 0.079455 | ns |
| RAB1A | 171.8163 | 183.83425 | 185.1212 | 0.01666 | * |
| RPN1 | 301.3054 | 337.06185 | 315.1476 | 0.642512 | ns |
| S100A10 | 179.7603 | 173.7956 | 207.4782 | 0.213523 | ns |
| SAT1 | 481.8684 | 598.08635 | 731.8453 | 9.4E-05 | *** |
| SEC61B | 296.6352 | 317.9616 | 324.2074 | 0.143969 | ns |
| SH3YL1 | 17.94719 | 19.48626 | 19.52053 | 0.461548 | ns |
| SNRPB2 | 44.10087 | 46.268135 | 51.03089 | 4.09E-05 | *** |
| SPINT2 | 359.7754 | 311.52195 | 295.3941 | 0.096434 | ns |
| SPOCK1 | 172.1813 | 237.8095 | 177.2168 | 0.580011 | ns |
| SRP9 | 233.8456 | 241.9098 | 250.0595 | 0.013966 | * |
| SURF4 | 316.4803 | 353.18005 | 335.6487 | 0.185513 | ns |
| TIMM17A | 37.45836 | 38.12566 | 41.13464 | 0.000795 | *** |
| TM9SF1 | 8.923855 | 9.987221 | 9.473416 | 0.249128 | ns |
| TMEM63A | 28.55497 | 33.881455 | 27.63865 | 0.9126 | ns |
| TNFSF10 | 186.4076 | 203.37205 | 163.7004 | 0.229949 | ns |
| TOMM5 | 13.64835 | 14.373695 | 16.00103 | 0.000265 | *** |
| UAP1 | 180.3617 | 162.98395 | 142.6859 | 0.517553 | ns |
| YWHAQ | 365.0688 | 416.4979 | 398.4457 | 0.195582 | ns |
| ALKBH7 | 147.5482 | 141.14145 | 138.1441 | 0.460158 | ns |
| AZGP1 | 2040.0697 | 1423.699 | 748.3615 | 8.52E-07 | *** |
| CBFA2T2 | 31.37373 | 30.53828 | 31.81556 | 0.796105 | ns |
| CCDC85B | 34.84439 | 29.64825 | 40.50449 | 0.057406 | ns |
| CD320 | 112.3994 | 117.1457 | 97.22971 | 0.000802 | *** |

| | | | | | |
|---|---|---|---|---|---|
| CDK5RAP3 | 68.12208 | 65.37259 | 72.89175 | 0.348488 | ns |
| CIRBP | 268.8148 | 234.013 | 247.2062 | 0.281475 | ns |
| DANCR | 98.7319 | 111.64115 | 107.3248 | 0.277409 | ns |
| EEF2 | 3013.859 | 3810.2255 | 3355.273 | 0.908974 | ns |
| EIF3B | 82.10126 | 89.76163 | 88.63491 | 0.075238 | ns |
| ERP29 | 318.9374 | 306.8329 | 260.0542 | 0.000319 | *** |
| FAM189A2 | 49.53572 | 45.20884 | 32.64895 | 9.85E-05 | *** |
| FAU | 659.0732 | 647.86555 | 684.3512 | 0.427506 | ns |
| FLNB | 142.676925 | 165.88835 | 166.7105 | 0.010097 | * |
| HSD17B6 | 93.99777 | 46.28713 | 30.76528 | 1.79E-05 | *** |
| IGFBP2 | 189.9743 | 184.7173 | 163.8747 | 0.028151 | * |
| KLK11 | 49.83298 | 90.49923 | 63.01987 | 0.735044 | ns |
| KLK2 | 3859.207 | 3265.866 | 2723.278 | 9.31E-05 | *** |
| MAP2K2 | 90.12834 | 89.8389 | 89.09514 | 0.592598 | ns |
| MICOS13 | 78.61293 | 81.06914 | 85.87733 | 0.210176 | ns |
| MZT2B | 147.5406 | 150.4899 | 144.6665 | 0.923489 | ns |
| NAA10 | 22.8018 | 22.238595 | 23.92125 | 0.717882 | ns |
| NACA | 258.759 | 275.2588 | 272.8958 | 0.401357 | ns |
| NDUFA11 | 37.08149 | 34.869165 | 34.81534 | 0.616529 | ns |
| NDUFB11 | 214.6506 | 214.6975 | 229.1601 | 0.139629 | ns |
| NISCH | 60.55577 | 61.49347 | 57.20259 | 0.156272 | ns |
| NUMA1 | 113.3564 | 120.95745 | 121.6881 | 0.03106 | * |
| PABPC1L | 7.19808 | 6.001395 | 9.244354 | 0.00318 | ** |
| PEBP1 | 917.62 | 887.5245 | 937.1257 | 0.220333 | ns |
| PILRB | 1.969798 | 1.8480905 | 2.773827 | 0.016764 | * |
| PIN1 | 50.33325 | 45.951715 | 47.81599 | 0.264472 | ns |
| PLEKHH1 | 33.88274 | 31.77592 | 28.462 | 0.919858 | ns |
| POLD4 | 38.65103 | 33.666205 | 34.06244 | 0.165836 | ns |
| RACK1 | 842.1544 | 905.74625 | 855.4255 | 0.918043 | ns |
| S100A13 | 22.06274 | 21.72928 | 21.06162 | 0.229063 | ns |
| SMS | 528.1361 | 407.7834 | 361.9013 | 0.409099 | ns |
| SND1 | 221.2092 | 230.35655 | 214.7126 | 0.943488 | ns |
| SNHG7 | 39.57774 | 41.32522 | 48.11984 | 0.007214 | ** |
| SORD | 201.4567 | 221.50855 | 194.9384 | 0.88004 | ns |
| TIMM13 | 123.8368 | 124.5225 | 132.677 | 0.244463 | ns |
| TM7SF2 | 131.3824 | 145.1476 | 155.746 | 0.019323 | * |
| VSTM2L | 115.0592 | 127.29475 | 118.1613 | 0.227297 | ns |

[a] ns $P > 0.05$; * $P < 0.05$; ** $P < 0.01$; ***$P < 0.001$.

**Supplementary Table 3. Expression levels of GS development related genes from patient 1 in TCGA-PRAD data stratified by pT. Related to Figure 3, 4.**

| Gene | pT = 2 (median) | pT = 3 (median) | pT = 4 (median) | P (pT = 2 vs. pT = 3) | P symbol[a] |
|---|---|---|---|---|---|
| ABCC4 | 279.2712 | 212.3662 | 245.9586 | 0.004238 | ** |
| ACSM1 | 64.77333 | 57.56744 | 118.9974 | 0.352776 | ns |
| ADIRF | 0 | 0 | 0 | 0.330502 | ns |
| AGR2 | 523.5504 | 497.4224 | 322.5492 | 0.629267 | ns |
| ANXA5 | 380.6523 | 416.6216 | 462.9429 | 0.004699 | ** |
| APLP2 | 500.6861 | 492.2861 | 582.409 | 0.498525 | ns |
| ARHGDIB | 86.99489 | 99.00518 | 124.2186 | 0.024475 | * |
| ARPC1B | 48.08293 | 54.92471 | 54.02797 | 0.001273 | ** |
| ASAH1 | 177.8803 | 157.1431 | 135.1895 | 0.009977 | ** |
| B4GALT1 | 100.2775 | 96.24144 | 87.86779 | 0.125756 | ns |
| CACYBP | 30.35413 | 31.51199 | 42.94532 | 0.011861 | * |
| CAPN2 | 47.03988 | 54.15117 | 81.84261 | 0.002931 | ** |
| CD46 | 134.1566 | 117.7486 | 140.408 | 0.023334 | * |
| CD59 | 154.4903 | 149.7601 | 156.3824 | 0.204629 | ns |
| CDK2AP1 | 71.84503 | 79.58808 | 79.78119 | 0.000356 | *** |
| CHMP5 | 117.4166 | 115.3814 | 139.9681 | 0.818344 | ns |
| CLTC | 126.2016 | 137.2363 | 187.2816 | 0.179677 | ns |
| CNIH4 | 14.82107 | 16.26475 | 17.03401 | 6.56E-07 | *** |
| COPB1 | 119.6818 | 123.4904 | 165.0574 | 0.37172 | ns |
| COPB2 | 100.7703 | 102.1829 | 131.2693 | 0.284768 | ns |
| CRIP2 | 33.44799 | 44.13673 | 43.10312 | 5.37E-06 | *** |
| DEGS1 | 205.8918 | 249.4837 | 401.9397 | 0.001663 | ** |
| DNAJA1 | 219.2702 | 220.9031 | 272.2513 | 0.360564 | ns |
| DSG2 | 67.10513 | 65.74605 | 73.90774 | 0.835258 | ns |
| EFR3A | 43.81617 | 42.61478 | 49.94501 | 0.897692 | ns |
| EIF4G2 | 272.0066 | 290.5204 | 325.2267 | 0.079731 | ns |
| EPCAM | 349.8178 | 361.8748 | 345.9612 | 0.581901 | ns |
| F5 | 10.16683 | 17.66429 | 69.03065 | 0.000318 | *** |
| FKBP5 | 99.25228 | 74.10269 | 67.69982 | 0.000391 | *** |
| GFPT1 | 98.9001 | 88.03092 | 107.7154 | 0.128461 | ns |
| GPR160 | 60.12946 | 68.77176 | 88.17149 | 0.008739 | ** |
| HEXB | 103.9693 | 90.19519 | 88.40009 | 0.004418 | ** |
| HNRNPC | 169.6468 | 182.1497 | 207.891 | 0.001186 | ** |
| HSP90AA1 | 590.2562 | 578.0276 | 739.9868 | 0.697686 | ns |
| HSPA5 | 707.3107 | 654.9221 | 748.6552 | 0.337864 | ns |
| HSPA8 | 734.3674 | 743.9394 | 892.7735 | 0.823093 | ns |

| | | | | | |
|---|---|---|---|---|---|
| HTATIP2 | 26.95586 | 25.82772 | 26.15816 | 0.871979 | ns |
| ISG15 | 71.1805 | 78.00758 | 102.5864 | 0.032961 | * |
| LAPTM4A | 475.8641 | 484.4175 | 500.4371 | 0.248859 | ns |
| LMAN1 | 111.8013 | 103.3929 | 121.1891 | 0.527367 | ns |
| LRP10 | 109.3614 | 108.9287 | 113.2381 | 0.381608 | ns |
| MANF | 142.4245 | 142.044 | 152.3841 | 0.952117 | ns |
| MANSC1 | 36.76127 | 32.37878 | 30.17716 | 0.031193 | * |
| MORF4L1 | 87.94628 | 92.96443 | 96.17187 | 0.020406 | * |
| MPC2 | 281.4312 | 265.3805 | 347.5952 | 0.233606 | ns |
| MPZL1 | 65.35348 | 65.11319 | 65.3052 | 0.745947 | ns |
| MT1E | 186.2828 | 113.325 | 13.55884 | 0.001893 | ** |
| MT1X | 117.0313 | 82.43262 | 52.35985 | 0.000171 | *** |
| MYL12A | 162.7847 | 171.1173 | 152.806 | 0.057234 | ns |
| MYL12B | 516.6895 | 547.5009 | 528.0228 | 0.003802 | ** |
| NANS | 149.1787 | 120.8572 | 99.82161 | 6.64E-06 | *** |
| PDIA4 | 394.9548 | 358.7799 | 357.4001 | 0.194875 | ns |
| PDXDC1 | 99.57226 | 100.5933 | 125.6803 | 0.420008 | ns |
| PIGR | 45.20575 | 35.97949 | 22.0277 | 0.411455 | ns |
| PPP1R1B | 160.9426 | 140.8073 | 51.38642 | 0.165377 | ns |
| PSMA6 | 9.743716 | 10.38223 | 13.93976 | 0.001876 | ** |
| PSME1 | 320.261 | 339.264 | 380.0295 | 0.09263 | ns |
| RAB1A | 185.571 | 182.329 | 215.6389 | 0.78843 | ns |
| RPN1 | 322.6588 | 321.9259 | 339.4741 | 0.467212 | ns |
| S100A10 | 165.0348 | 204.2217 | 203.6454 | 0.002371 | ** |
| SAT1 | 574.4774 | 659.8346 | 794.3119 | 0.003418 | ** |
| SEC61B | 315.5217 | 323.4652 | 308.241 | 0.281421 | ns |
| SH3YL1 | 17.87534 | 20.53164 | 18.28575 | 0.092498 | ns |
| SNRPB2 | 44.28816 | 50.18282 | 53.71785 | 1.77E-05 | *** |
| SPINT2 | 330.2436 | 299.869 | 262.4512 | 0.005819 | ** |
| SPOCK1 | 234.0392 | 199.2054 | 170.1818 | 0.072331 | ns |
| SRP9 | 235.6318 | 250.531 | 270.713 | 0.013533 | * |
| SURF4 | 342.2565 | 338.7274 | 420.7047 | 0.695679 | ns |
| TIMM17A | 38.98065 | 39.75401 | 42.85137 | 0.014322 | * |
| TM9SF1 | 10.00511 | 9.517 | 7.922345 | 0.181444 | ns |
| TMEM63A | 33.28831 | 29.79358 | 18.30852 | 0.449937 | ns |
| TNFSF10 | 199.3018 | 181.8078 | 134.3671 | 0.113508 | ns |
| TOMM5 | 14.35031 | 15.15142 | 17.23783 | 0.002673 | ** |
| UAP1 | 168.122 | 146.7927 | 171.4676 | 0.126091 | ns |
| YWHAQ | 404.3644 | 403.3621 | 469.0056 | 0.812549 | ns |
| ALKBH7 | 142.1263 | 138.3135 | 140.0363 | 0.187275 | ns |
| AZGP1 | 1804.101 | 1013.28 | 319.8476 | 1.62E-11 | *** |
| CBFA2T2 | 30.71837 | 30.80044 | 41.77784 | 0.891252 | ns |
| CCDC85B | 29.69212 | 36.74847 | 38.08501 | 0.038663 | * |
| CD320 | 119.8897 | 104.197 | 112.7139 | 5.47E-05 | *** |

| | | | | | |
|---|---|---|---|---|---|
| CDK5RAP3 | 68.7004 | 70.49347 | 51.80364 | 0.569404 | ns |
| CIRBP | 248.2864 | 235.4683 | 168.8106 | 0.341295 | ns |
| DANCR | 111.6929 | 107.5336 | 90.20408 | 0.965634 | ns |
| EEF2 | 3758.035 | 3477.452 | 3649.123 | 0.278401 | ns |
| EIF3B | 87.8597 | 89.74496 | 87.50856 | 0.385311 | ns |
| ERP29 | 315.1747 | 270.5474 | 258.4853 | 1.8E-05 | *** |
| FAM189A2 | 48.52823 | 37.3766 | 16.37562 | 1.56E-07 | *** |
| FAU | 649.8077 | 673.6505 | 620.9704 | 0.441035 | ns |
| FLNB | 163.2987 | 164.512 | 244.2139 | 0.488675 | ns |
| HSD17B6 | 54.89847 | 34.93166 | 40.67009 | 0.003344 | ** |
| IGFBP2 | 189.9743 | 170.6243 | 154.4874 | 0.038727 | * |
| KLK11 | 80.93517 | 69.236 | 101.8112 | 0.700198 | ns |
| KLK2 | 3553.962 | 2900.157 | 1708.084 | 7.6E-07 | *** |
| MAP2K2 | 90.4062 | 89.51909 | 86.95886 | 0.172736 | ns |
| MICOS13 | 81.17424 | 83.02705 | 103.1036 | 0.158257 | ns |
| MZT2B | 148.6407 | 146.9057 | 150.8642 | 0.766065 | ns |
| NAA10 | 22.22474 | 23.81165 | 24.40712 | 0.085614 | ns |
| NACA | 273.6942 | 273.794 | 254.1789 | 0.32972 | ns |
| NDUFA11 | 34.61533 | 35.24819 | 34.67196 | 0.874117 | ns |
| NDUFB11 | 213.3522 | 227.0551 | 237.459 | 0.090931 | ns |
| NISCH | 62.29552 | 57.56577 | 51.13203 | 0.001867 | ** |
| NUMA1 | 119.9577 | 121.134 | 134.5715 | 0.266251 | ns |
| PABPC1L | 6.857247 | 7.726055 | 5.836041 | 0.036972 | * |
| PEBP1 | 914.7733 | 900.6396 | 992.2793 | 0.988902 | ns |
| PILRB | 1.932882 | 2.387009 | 3.672808 | 0.0226 | * |
| PIN1 | 48.25212 | 46.63177 | 51.72013 | 0.168083 | ns |
| PLEKHH1 | 31.6592 | 30.01904 | 30.4175 | 0.727529 | ns |
| POLD4 | 35.40669 | 33.33004 | 29.08868 | 0.058578 | ns |
| RACK1 | 879.4071 | 884.6452 | 896.8996 | 0.851716 | ns |
| S100A13 | 20.68619 | 21.83039 | 21.60767 | 0.273616 | ns |
| SMS | 475.5499 | 365.044 | 368.0414 | 0.003329 | ** |
| SND1 | 230.64 | 222.0639 | 213.2914 | 0.282939 | ns |
| SNHG7 | 40.37838 | 46.37532 | 35.58706 | 0.00483 | ** |
| SORD | 223.6039 | 207.0964 | 165.4806 | 0.04102 | * |
| TIMM13 | 123.379 | 130.9155 | 130.8561 | 0.313165 | ns |
| TM7SF2 | 146.0656 | 147.9036 | 132.8352 | 0.460588 | ns |
| VSTM2L | 117.859 | 129.142 | 222.9158 | 0.195577 | ns |

[a] ns P > 0.05; * P < 0.05; ** P < 0.01; ***P < 0.001.

**Supplementary Table 4. Expression levels of GS development related genes from patient 1 in TCGA-PRAD data stratified by TP53 mutation. Related to Figure 3, 4.**

| Gene | Wild type (median) | Mutation (median) | P (wild vs. mut) | P symbol[a] |
|---|---|---|---|---|
| ABCC4 | 2.59E+02 | 182.76925 | 0.057984971 | ns |
| ACSM1 | 67.44041 | 60.969795 | 0.340530697 | ns |
| ADIRF | 0 | 0 | 0.46908958 | ns |
| AGR2 | 509.8044 | 503.872 | 0.976902453 | ns |
| ANXA5 | 403.2229 | 440.77225 | 0.080337246 | ns |
| APLP2 | 496.8398 | 502.924 | 0.660719279 | ns |
| ARHGDIB | 94.05682 | 98.44227 | 0.177648843 | ns |
| ARPC1B | 49.94487 | 54.8233 | 0.109232187 | ns |
| ASAH1 | 163.9571 | 150.0145 | 0.641532889 | ns |
| B4GALT1 | 95.60861 | 87.13802 | 0.532485423 | ns |
| CACYBP | 31.22633 | 32.63064 | 0.127204451 | ns |
| CAPN2 | 51.13824 | 57.43833 | 0.024881226 | * |
| CD46 | 121.9074 | 124.206 | 0.778389382 | ns |
| CD59 | 150.2751 | 135.50195 | 0.394012984 | ns |
| CDK2AP1 | 75.01727 | 107.6078 | 6.61686E-05 | *** |
| CHMP5 | 116.5497 | 112.72315 | 0.746611784 | ns |
| CLTC | 131.5749 | 157.4651 | 0.059221326 | ns |
| CNIH4 | 15.65596 | 17.245565 | 0.010803835 | * |
| COPB1 | 120.1814 | 146.95025 | 0.008508253 | ** |
| COPB2 | 101.1326 | 115.33915 | 0.057679265 | ns |
| CRIP2 | 38.84864 | 55.456185 | 0.00250577 | ** |
| DEGS1 | 227.8885 | 284.29465 | 0.009741244 | ** |
| DNAJA1 | 219.073 | 242.7743 | 0.26723143 | ns |
| DSG2 | 63.25797 | 83.8134 | 0.11418707 | ns |
| EFR3A | 42.27505 | 51.64491 | 0.092429139 | ns |
| EIF4G2 | 278.5122 | 339.66375 | 0.002984099 | ** |
| EPCAM | 362.2263 | 415.00485 | 0.080337246 | ns |
| F5 | 14.26411 | 20.749735 | 0.204754855 | ns |
| FKBP5 | 82.09173 | 70.82538 | 0.231120874 | ns |
| GFPT1 | 93.40384 | 92.87582 | 0.724791219 | ns |
| GPR160 | 66.00727 | 96.780245 | 0.001425633 | ** |
| HEXB | 92.9629 | 80.163235 | 0.028442585 | * |
| HNRNPC | 178.2365 | 189.13135 | 0.014172624 | * |
| HSP90AA1 | 584.6578 | 643.8479 | 0.212721531 | ns |

| | | | | |
|---|---|---|---|---|
| HSPA5 | 685.0906 | 716.50015 | 0.871200213 | ns |
| HSPA8 | 741.6467 | 824.195 | 0.261777434 | ns |
| HTATIP2 | 25.31049 | 30.853045 | 0.213999615 | ns |
| ISG15 | 76.03515 | 64.095785 | 0.234749998 | ns |
| LAPTM4A | 476.9992 | 507.1801 | 0.080939911 | ns |
| LMAN1 | 106.8154 | 108.0513 | 0.701474017 | ns |
| LRP10 | 107.8395 | 111.3372 | 0.760685609 | ns |
| MANF | 143.8878 | 139.0091 | 0.422896672 | ns |
| MANSC1 | 33.4789 | 32.051375 | 0.827644939 | ns |
| MORF4L1 | 89.06578 | 98.59012 | 0.020484115 | * |
| MPC2 | 277.7331 | 263.3951 | 0.18406517 | ns |
| MPZL1 | 65.07713 | 67.67533 | 0.332380263 | ns |
| MT1E | 146.5664 | 49.348225 | 3.07276E-05 | *** |
| MT1X | 93.4244 | 56.328675 | 0.000138881 | *** |
| MYL12A | 168.6132 | 181.01575 | 0.433699737 | ns |
| MYL12B | 529.1234 | 557.86455 | 0.849365098 | ns |
| NANS | 138.305 | 97.540315 | 1.7167E-05 | *** |
| PDIA4 | 379.7176 | 381.26815 | 0.884898284 | ns |
| PDXDC1 | 100.1014 | 109.82865 | 0.176163615 | ns |
| PIGR | 35.67488 | 43.4048 | 0.547032654 | ns |
| PPP1R1B | 154.8121 | 103.1148 | 0.188307383 | ns |
| PSMA6 | 9.9176 | 11.74959 | 0.00170514 | ** |
| PSME1 | 334.8019 | 332.70865 | 0.922503648 | ns |
| RAB1A | 182.2567 | 200.045 | 0.063226664 | ns |
| RPN1 | 322.6588 | 324.5808 | 0.803367176 | ns |
| S100A10 | 182.8361 | 157.9337 | 0.760685609 | ns |
| SAT1 | 616.5136 | 706.12485 | 0.029549882 | * |
| SEC61B | 324.3105 | 322.70275 | 0.996303889 | ns |
| SH3YL1 | 18.99555 | 20.651605 | 0.47775625 | ns |
| SNRPB2 | 47.87549 | 55.914575 | 0.000183533 | *** |
| SPINT2 | 307.7005 | 298.16395 | 0.295134918 | ns |
| SPOCK1 | 221.0967 | 174.0692 | 0.100551186 | ns |
| SRP9 | 242.3478 | 266.9014 | 0.008110877 | ** |
| SURF4 | 339.3735 | 373.4456 | 0.148696198 | ns |
| TIMM17A | 39.14292 | 41.30848 | 0.28982555 | ns |
| TM9SF1 | 9.628334 | 9.875317 | 0.600653004 | ns |
| TMEM63A | 29.55643 | 30.971525 | 0.777501371 | ns |
| TNFSF10 | 187.4799 | 172.40975 | 0.096770307 | ns |
| TOMM5 | 14.83574 | 15.67612 | 0.146751822 | ns |
| UAP1 | 157.0983 | 163.5553 | 0.881241926 | ns |
| YWHAQ | 403.4328 | 480.8308 | 0.006871511 | ** |
| ALKBH7 | 142.206 | 119.1344 | 0.000642699 | *** |
| AZGP1 | 1302.059 | 347.65255 | 1.33874E-06 | *** |
| CBFA2T2 | 30.56993 | 32.791085 | 0.159743512 | ns |

| | | | | |
|---|---|---|---|---|
| CCDC85B | 34.86199 | 32.510775 | 0.69975763 | ns |
| CD320 | 110.1273 | 84.471195 | 0.000321832 | *** |
| CDK5RAP3 | 68.64726 | 66.353225 | 0.307043581 | ns |
| CIRBP | 243.1457 | 221.2029 | 0.136708721 | ns |
| DANCR | 113.0564 | 101.90485 | 0.095152332 | ns |
| EEF2 | 3727.116 | 2957.75 | 0.005464675 | ** |
| EIF3B | 88.76513 | 94.585675 | 0.171038201 | ns |
| ERP29 | 296.8072 | 235.99235 | 0.000133767 | *** |
| FAM189A2 | 41.15724 | 35.38751 | 0.223982584 | ns |
| FAU | 663.7564 | 690.85065 | 0.943680957 | ns |
| FLNB | 159.2574 | 171.19545 | 0.185215056 | ns |
| HSD17B6 | 39.6149 | 26.84959 | 0.004682769 | ** |
| IGFBP2 | 184.5068 | 132.65825 | 7.1186E-05 | *** |
| KLK11 | 69.32022 | 80.470205 | 0.824938806 | ns |
| KLK2 | 3210.842 | 2561.4025 | 0.00015391 | *** |
| MAP2K2 | 90.06863 | 84.80277 | 0.010625768 | * |
| MICOS13 | 83.95702 | 80.470315 | 0.160777804 | ns |
| MZT2B | 151.1739 | 136.19425 | 0.170676419 | ns |
| NAA10 | 23.50898 | 22.06937 | 0.237498219 | ns |
| NACA | 276.4342 | 268.3965 | 0.652349002 | ns |
| NDUFA11 | 35.29549 | 31.619555 | 0.007756847 | ** |
| NDUFB11 | 221.9845 | 226.1881 | 0.617679656 | ns |
| NISCH | 59.09335 | 49.33461 | 0.024363473 | * |
| NUMA1 | 119.8715 | 136.27505 | 0.01992362 | * |
| PABPC1L | 7.196652 | 8.1080705 | 0.462099461 | ns |
| PEBP1 | 904.2461 | 910.84025 | 0.794422011 | ns |
| PILRB | 2.096697 | 3.057003 | 0.016361799 | * |
| PIN1 | 48.13693 | 40.79787 | 0.002873537 | ** |
| PLEKHH1 | 31.28044 | 26.56988 | 0.73175049 | ns |
| POLD4 | 34.14616 | 30.728345 | 0.006895488 | ** |
| RACK1 | 902.6517 | 807.04805 | 0.131890262 | ns |
| S100A13 | 21.23058 | 19.984125 | 0.336439602 | ns |
| SMS | 398.5283 | 413.4065 | 0.455078532 | ns |
| SND1 | 227.5617 | 211.4558 | 0.20766432 | ns |
| SNHG7 | 45.58529 | 38.74563 | 0.165671495 | ns |
| SORD | 215.8933 | 178.9575 | 0.033001578 | * |
| TIMM13 | 128.3988 | 132.9152 | 0.443971 | ns |
| TM7SF2 | 144.6104 | 150.22885 | 0.909638994 | ns |
| VSTM2L | 121.2175 | 165.49105 | 0.072092903 | ns |

[a] ns P > 0.05; * P < 0.05; ** P < 0.01; ***P < 0.001.

**Supplementary Table 5. Survival analysis and univariate Cox regression analysis of GS development related genes from patient 1 in RFS outcomes. Related to Figure 3, 4.**

| Gene | HR | HR.95L | HR.95H | P value | KM |
|---|---|---|---|---|---|
| ABCC4 | 1.000032593 | 0.998960403 | 1.001105933 | 0.952515093 | 0.180848663 |
| ACSM1 | 1.000137117 | 0.998390528 | 1.001886762 | 0.877811129 | 0.231360664 |
| AGR2 | 0.999979748 | 0.999494371 | 1.000465361 | 0.934839995 | 0.042218836 |
| ANXA5 | 1.001143526 | 0.999727641 | 1.002561416 | 0.113482943 | 0.011634285 |
| APLP2 | 0.99926286 | 0.998003835 | 1.000523474 | 0.251636886 | 0.179882523 |
| ARHGDIB | 1.002052327 | 0.999776275 | 1.004333559 | 0.077209549 | 0.008788143 |
| ARPC1B | 1.009966787 | 1.004488631 | 1.01547482 | 0.000351728 | 9.22E-05 |
| ASAH1 | 0.998868557 | 0.995863539 | 1.001882643 | 0.461466702 | 0.069401481 |
| B4GALT1 | 0.998371952 | 0.992922244 | 1.003851572 | 0.559593683 | 0.070439904 |
| CACYBP | 1.008618907 | 0.986452191 | 1.031283734 | 0.449103479 | 0.069309151 |
| CAPN2 | 1.004950845 | 0.997284812 | 1.012675806 | 0.206209241 | 0.015302411 |
| CD46 | 0.997228561 | 0.992883754 | 1.00159238 | 0.212854589 | 0.019308203 |
| CD59 | 0.997908439 | 0.993155137 | 1.00268449 | 0.390079605 | 0.08744171 |
| CDK2AP1 | 1.003677254 | 0.997915788 | 1.009471985 | 0.211429923 | 0.025506427 |
| CHMP5 | 1.003020161 | 0.996625318 | 1.009456036 | 0.355438873 | 0.078024307 |
| CLTC | 0.999899662 | 0.996158019 | 1.00365536 | 0.958163487 | 0.287402344 |
| CNIH4 | 1.047887786 | 1.013888991 | 1.083026664 | 0.005442341 | 7.07E-05 |
| COPB1 | 1.001325734 | 0.996522597 | 1.006152021 | 0.589171634 | 0.199532393 |
| COPB2 | 0.999845431 | 0.996468938 | 1.003233366 | 0.928633397 | 0.154393797 |
| CRIP2 | 1.003447903 | 1.001330663 | 1.00556962 | 0.001403638 | 1.80E-07 |
| DEGS1 | 1.000618385 | 0.999959118 | 1.001278087 | 0.06600539 | 0.000276742 |
| DNAJA1 | 1.001986851 | 0.999368233 | 1.004612331 | 0.13711152 | 0.017010106 |
| DSG2 | 0.998917597 | 0.993697877 | 1.004164735 | 0.685366032 | 0.124886337 |
| EFR3A | 0.995945769 | 0.986672196 | 1.005306502 | 0.394695677 | 0.046869126 |
| EIF4G2 | 1.000494127 | 0.998181972 | 1.002811639 | 0.675596172 | 0.083556851 |
| EPCAM | 1.000086558 | 0.998837085 | 1.001337595 | 0.892058936 | 0.258180878 |
| F5 | 1.003372666 | 1.000217707 | 1.006537576 | 0.036132011 | 0.005557783 |
| FKBP5 | 0.996832924 | 0.992127711 | 1.001560453 | 0.188830089 | 0.05145723 |
| GFPT1 | 0.997904837 | 0.992483389 | 1.0033559 | 0.450493301 | 0.060534335 |
| GPR160 | 1.000606398 | 0.995269882 | 1.005971529 | 0.824168084 | 0.050361271 |
| HEXB | 0.999031141 | 0.994819753 | 1.003260358 | 0.652902294 | 0.048107725 |
| HNRNPC | 1.002930038 | 0.997787672 | 1.008098908 | 0.264627227 | 0.002393211 |
| HSP90AA1 | 1.000406009 | 0.999569069 | 1.00124365 | 0.341809919 | 0.101971608 |
| HSPA5 | 1.000115047 | 0.999314719 | 1.000916017 | 0.778212817 | 0.023002226 |
| HSPA8 | 1.000458314 | 0.999753954 | 1.00116317 | 0.202254487 | 0.024855954 |

| | | | | | |
|---|---|---|---|---|---|
| HTATIP2 | 0.990910403 | 0.972107302 | 1.010077205 | 0.350216702 | 0.064546696 |
| ISG15 | 1.000392721 | 1.00001955 | 1.000766031 | 0.039144731 | 0.011502037 |
| LAPTM4A | 1.000073635 | 0.99799426 | 1.002157343 | 0.944721393 | 0.161134095 |
| LMAN1 | 0.997284881 | 0.992885772 | 1.001703481 | 0.228058721 | 0.094483797 |
| LRP10 | 1.0018586 | 0.99578675 | 1.007967473 | 0.549384517 | 0.18566262 |
| MANF | 1.002151741 | 0.998084083 | 1.006235977 | 0.300293824 | 0.001937018 |
| MANSC1 | 0.98995863 | 0.974610049 | 1.005548926 | 0.205557763 | 0.000346194 |
| MORF4L1 | 1.004241999 | 0.99506822 | 1.013500353 | 0.365962719 | 0.106191643 |
| MPC2 | 0.999804603 | 0.998060848 | 1.001551404 | 0.826329502 | 0.098883163 |
| MPZL1 | 1.00563256 | 0.994544312 | 1.016844432 | 0.32075961 | 0.035777112 |
| MT1E | 0.996783433 | 0.994910925 | 0.998659465 | 0.000784464 | 2.08E-05 |
| MT1X | 0.994991644 | 0.991434043 | 0.998562011 | 0.006007468 | 1.44E-05 |
| MYL12A | 0.99972948 | 0.995520431 | 1.003956324 | 0.899979898 | 0.180689158 |
| MYL12B | 1.000261929 | 0.998540981 | 1.001985843 | 0.765634792 | 0.01769465 |
| NANS | 0.989370994 | 0.984073352 | 0.994697155 | 9.58E-05 | 1.97E-05 |
| PDIA4 | 1.000569758 | 0.99933234 | 1.001808708 | 0.366979385 | 0.038055179 |
| PDXDC1 | 1.00220766 | 0.995709957 | 1.008747766 | 0.506377615 | 0.004231705 |
| PIGR | 0.99963852 | 0.998596337 | 1.000681791 | 0.496926305 | 0.099362215 |
| PPP1R1B | 0.998179517 | 0.996071763 | 1.00029173 | 0.091122078 | 0.001102067 |
| PSMA6 | 1.051899816 | 0.984782736 | 1.123591206 | 0.132549652 | 0.027257535 |
| PSME1 | 1.001477848 | 0.998956566 | 1.004005493 | 0.250872768 | 0.018547745 |
| RAB1A | 1.002158652 | 0.99753866 | 1.00680004 | 0.360375672 | 0.04111419 |
| RPN1 | 0.999553593 | 0.997631648 | 1.001479242 | 0.649326659 | 0.033592503 |
| S100A10 | 1.000894514 | 0.999968724 | 1.001821162 | 0.05826196 | 0.040941955 |
| SAT1 | 1.000444221 | 1.000094087 | 1.000794476 | 0.012891278 | 0.001704526 |
| SEC61B | 0.999897929 | 0.997355889 | 1.002446447 | 0.937354692 | 0.094591029 |
| SH3YL1 | 1.004442212 | 0.985832505 | 1.023403218 | 0.642266821 | 0.045865957 |
| SNRPB2 | 1.006784601 | 0.990957112 | 1.022864885 | 0.402954272 | 0.085471554 |
| SPINT2 | 0.998911728 | 0.996461014 | 1.00136847 | 0.384952889 | 0.106703511 |
| SPOCK1 | 0.999208761 | 0.997957019 | 1.000462074 | 0.215847142 | 0.079988995 |
| SRP9 | 1.00024878 | 0.997022254 | 1.003485748 | 0.880056766 | 0.256518892 |
| SURF4 | 1.000498052 | 0.998120731 | 1.002881035 | 0.681637887 | 0.120709904 |
| TIMM17A | 1.017502866 | 0.999780884 | 1.035538985 | 0.052926597 | 5.11E-05 |
| TM9SF1 | 1.014419712 | 0.94968846 | 1.083563079 | 0.670431772 | 0.132371414 |
| TMEM63A | 1.002710717 | 0.991836817 | 1.013703831 | 0.626544255 | 0.060894623 |
| TNFSF10 | 0.99815561 | 0.99565373 | 1.000663777 | 0.149374344 | 0.027965541 |
| TOMM5 | 1.035347483 | 0.985267748 | 1.087972697 | 0.169679815 | 0.019110599 |
| UAP1 | 0.999371269 | 0.996220242 | 1.002532262 | 0.696287813 | 0.120877533 |
| YWHAQ | 1.000229777 | 0.998441094 | 1.002021663 | 0.801363042 | 0.142955115 |
| ALKBH7 | 0.999166271 | 0.996004329 | 1.002338251 | 0.60602021 | 0.155901211 |
| AZGP1 | 0.999651708 | 0.999383757 | 0.99991973 | 0.010869925 | 0.000103045 |
| CBFA2T2 | 1.004823286 | 0.990219857 | 1.019642082 | 0.519460338 | 0.043036675 |
| CCDC85B | 1.000574487 | 0.994180574 | 1.007009522 | 0.860617517 | 0.01116393 |
| CD320 | 0.994707518 | 0.989397698 | 1.000045834 | 0.051994351 | 0.011473513 |

| | | | | | |
|---|---|---|---|---|---|
| CDK5RAP3 | 1.008809045 | 1.001938664 | 1.015726537 | 0.011887965 | 0.00038203 |
| CIRBP | 1.001699092 | 0.998532048 | 1.004876181 | 0.293381566 | 0.074086145 |
| DANCR | 0.996569102 | 0.991970697 | 1.001188823 | 0.145265699 | 0.07770942 |
| EEF2 | 0.999954753 | 0.999776451 | 1.000133086 | 0.61896403 | 0.107226546 |
| EIF3B | 1.001375364 | 0.992768564 | 1.010056781 | 0.754988749 | 0.02270614 |
| ERP29 | 0.996877488 | 0.994203092 | 0.999559078 | 0.022505378 | 3.86E-05 |
| FAM189A2 | 0.986362362 | 0.974853374 | 0.998007225 | 0.021843523 | 0.002861861 |
| FAU | 0.999691354 | 0.99862836 | 1.000755479 | 0.56956235 | 0.199467353 |
| FLNB | 1.001103858 | 0.998072568 | 1.004144355 | 0.475819833 | 0.017241302 |
| HSD17B6 | 0.997038317 | 0.994210439 | 0.99987424 | 0.040682563 | 0.0018727 |
| IGFBP2 | 0.998744008 | 0.996580138 | 1.000912576 | 0.256085905 | 0.032738212 |
| KLK11 | 1.000658655 | 0.999838717 | 1.001479265 | 0.11541618 | 0.020676252 |
| KLK2 | 0.999975686 | 0.999781946 | 1.000169464 | 0.805723907 | 0.195685032 |
| MAP2K2 | 1.000558524 | 0.992499356 | 1.008683133 | 0.892357915 | 0.211982838 |
| MICOS13 | 1.00083887 | 0.994296109 | 1.007424684 | 0.802141551 | 0.079144989 |
| MZT2B | 0.999940273 | 0.99742755 | 1.002459325 | 0.962889136 | 0.086690916 |
| NAA10 | 1.005128225 | 0.986697866 | 1.023902843 | 0.588007684 | 0.001863312 |
| NACA | 0.998518629 | 0.99555886 | 1.001487197 | 0.32768568 | 0.142597229 |
| NDUFA11 | 0.995666161 | 0.98258784 | 1.008918555 | 0.519698935 | 0.209759592 |
| NDUFB11 | 0.999882413 | 0.997236515 | 1.002535332 | 0.930685304 | 0.059808466 |
| NISCH | 1.007062341 | 0.995176478 | 1.019090163 | 0.245331525 | 0.042073218 |
| NUMA1 | 1.002447199 | 0.997235567 | 1.007686067 | 0.358065241 | 0.026100571 |
| PABPC1L | 1.052184091 | 1.028552866 | 1.076358249 | 1.14E-05 | 7.22E-08 |
| PEBP1 | 0.999688777 | 0.998532167 | 1.000846726 | 0.598190407 | 0.000314995 |
| PILRB | 1.087582118 | 1.049927193 | 1.126587512 | 3.01E-06 | 3.44E-07 |
| PIN1 | 1.000112414 | 0.986009115 | 1.014417439 | 0.987623019 | 0.088046845 |
| PLEKHH1 | 1.000365802 | 0.99149708 | 1.009313852 | 0.935841937 | 0.084295953 |
| POLD4 | 0.989627633 | 0.970944172 | 1.008670611 | 0.283636216 | 0.065390984 |
| RACK1 | 0.999409117 | 0.99850931 | 1.000309735 | 0.198406919 | 0.085813516 |
| S100A13 | 0.996765308 | 0.980355591 | 1.013449701 | 0.702061117 | 0.032441021 |
| SMS | 0.999989723 | 0.999664673 | 1.000314879 | 0.95059892 | 0.198210321 |
| SND1 | 0.999578034 | 0.996182013 | 1.002985631 | 0.80795386 | 0.200478518 |
| SNHG7 | 1.005071157 | 0.996992344 | 1.013215434 | 0.219281586 | 0.006402366 |
| SORD | 0.999692852 | 0.997876581 | 1.001512427 | 0.740571651 | 0.304757239 |
| TIMM13 | 0.999891878 | 0.995744933 | 1.004056093 | 0.959331304 | 0.010585831 |
| TM7SF2 | 0.999962387 | 0.996771647 | 1.00316334 | 0.981596569 | 0.087131099 |
| VSTM2L | 1.000277918 | 0.999543428 | 1.001012947 | 0.458423194 | 0.091766643 |

HR: hazard ratio; HR.95L: low value of HR 95% confidence interval; HR.95H: high value of HR 95% confidence interval; P value: P value of Cox regression analysis; KM: P value of Kaplan-Meier analysis.

**Supplementary Table 6. Survival analysis and univariate Cox regression analysis of GS development related genes from patient 1 in PFS outcomes. Related to Figure 3, 4.**

| Gene | HR | HR.95L | HR.95H | P value | KM |
|---|---|---|---|---|---|
| ABCC4 | 0.999381092 | 0.998448218 | 1.000314838 | 0.193836313 | 0.026992178 |
| ACSM1 | 0.999691788 | 0.998211542 | 1.001174229 | 0.683470061 | 0.044135736 |
| AGR2 | 0.999693884 | 0.999261208 | 1.000126746 | 0.165699503 | 0.001394389 |
| ANXA5 | 1.000597449 | 0.999445313 | 1.001750913 | 0.309594661 | 0.100959941 |
| APLP2 | 0.999255268 | 0.998262192 | 1.000249332 | 0.141955575 | 0.003688303 |
| ARHGDIB | 0.999866798 | 0.99781596 | 1.001921851 | 0.89881324 | 0.020761723 |
| ARPC1B | 1.0048728 | 0.99966816 | 1.010104539 | 0.066550855 | 0.000945965 |
| ASAH1 | 0.998266138 | 0.995778284 | 1.000760207 | 0.172859371 | 0.007161131 |
| B4GALT1 | 0.994959942 | 0.990333285 | 0.999608213 | 0.033607822 | 0.001714215 |
| CACYBP | 1.011760598 | 0.995096058 | 1.028704214 | 0.167645249 | 0.052603292 |
| CAPN2 | 1.003515266 | 0.997361043 | 1.009707463 | 0.263547306 | 0.022398488 |
| CD46 | 0.995819885 | 0.992311842 | 0.999340328 | 0.01999403 | 0.001283365 |
| CD59 | 0.996077729 | 0.992284782 | 0.999885174 | 0.043491691 | 0.007127815 |
| CDK2AP1 | 1.001966587 | 0.997164524 | 1.006791775 | 0.422827751 | 0.043397377 |
| CHMP5 | 1.000457485 | 0.995165925 | 1.005777181 | 0.865765283 | 0.171780433 |
| CLTC | 0.998829657 | 0.995812353 | 1.001856103 | 0.448072813 | 0.068929129 |
| CNIH4 | 1.065188205 | 1.036403489 | 1.094772378 | 6.24E-06 | 5.33E-05 |
| COPB1 | 0.999734327 | 0.995868098 | 1.003615567 | 0.893084057 | 0.040418098 |
| COPB2 | 0.998987296 | 0.995596623 | 1.002389516 | 0.559153483 | 0.111617411 |
| CRIP2 | 1.006356467 | 1.004047485 | 1.008670758 | 6.43E-08 | 9.27E-06 |
| DEGS1 | 1.000947352 | 1.000432779 | 1.00146219 | 0.000307186 | 1.13E-05 |
| DNAJA1 | 1.000966444 | 0.998766314 | 1.003171421 | 0.389559849 | 0.010677887 |
| DSG2 | 0.996530435 | 0.992140614 | 1.00093968 | 0.122833343 | 0.012183435 |
| EFR3A | 0.991785332 | 0.983822686 | 0.999812424 | 0.044901534 | 0.002618061 |
| EIF4G2 | 0.999804065 | 0.997954416 | 1.001657142 | 0.835690772 | 0.119190595 |
| EPCAM | 1.000231659 | 0.999257704 | 1.001206564 | 0.641206676 | 0.171941735 |
| F5 | 1.000220206 | 0.996716908 | 1.003735818 | 0.902111302 | 0.058486259 |
| FKBP5 | 0.995549629 | 0.991659335 | 0.999455186 | 0.025564466 | 0.005095742 |
| GFPT1 | 0.996307447 | 0.991903529 | 1.000730917 | 0.101692018 | 0.012313662 |
| GPR160 | 1.000564508 | 0.996357556 | 1.004789224 | 0.792922205 | 0.233550839 |
| HEXB | 0.997513022 | 0.99377263 | 1.001267491 | 0.193905325 | 0.003039735 |
| HNRNPC | 1.003961827 | 0.99989124 | 1.008048986 | 0.056456751 | 0.00040234 |
| HSP90AA1 | 1.00009142 | 0.999376528 | 1.000806823 | 0.802153551 | 0.281771466 |
| HSPA5 | 0.999852306 | 0.999199992 | 1.000505047 | 0.657341033 | 0.116823214 |
| HSPA8 | 1.00026397 | 0.999699025 | 1.000829234 | 0.359849405 | 0.055496766 |

| | | | | | |
|---|---|---|---|---|---|
| HTATIP2 | 0.986364434 | 0.971397599 | 1.001561871 | 0.078422949 | 0.032272851 |
| ISG15 | 1.000370501 | 1.000081877 | 1.000659209 | 0.011867251 | 0.005587959 |
| LAPTM4A | 0.999751138 | 0.998129033 | 1.00137588 | 0.763861336 | 0.017318648 |
| LMAN1 | 0.99669073 | 0.993194834 | 1.000198932 | 0.06445655 | 0.011403055 |
| LRP10 | 0.999347433 | 0.994493593 | 1.004224963 | 0.792721568 | 0.376230057 |
| MANF | 1.00305887 | 0.999863857 | 1.006264092 | 0.060611322 | 0.010026013 |
| MANSC1 | 0.982671317 | 0.970118589 | 0.995386469 | 0.007700491 | 2.60E-05 |
| MORF4L1 | 1.004999786 | 0.997755391 | 1.012296779 | 0.176641034 | 0.037161686 |
| MPC2 | 1.000404189 | 0.999133871 | 1.001676123 | 0.533054569 | 0.021356398 |
| MPZL1 | 0.996268389 | 0.986917708 | 1.005707666 | 0.437135627 | 0.015617637 |
| MT1E | 0.999701311 | 0.998995371 | 1.00040775 | 0.407183276 | 0.00036066 |
| MT1X | 0.999075991 | 0.997191163 | 1.000964382 | 0.337310679 | 4.36E-05 |
| MYL12A | 1.000002566 | 0.99676297 | 1.003252691 | 0.998763479 | 0.29254182 |
| MYL12B | 1.000862352 | 0.999539629 | 1.002186825 | 0.201422821 | 0.005530457 |
| NANS | 0.995113921 | 0.991516033 | 0.998724864 | 0.008039765 | 0.001530125 |
| PDIA4 | 0.999516701 | 0.998434112 | 1.000600465 | 0.381955876 | 0.093757068 |
| PDXDC1 | 0.999487968 | 0.994293159 | 1.004709918 | 0.847245474 | 0.051216762 |
| PIGR | 0.99931589 | 0.998360716 | 1.000271977 | 0.160734542 | 0.02388183 |
| PPP1R1B | 0.998731737 | 0.997166524 | 1.000299407 | 0.112768712 | 0.002411486 |
| PSMA6 | 1.052728867 | 0.99991491 | 1.108332375 | 0.050379963 | 0.005159918 |
| PSME1 | 1.001801913 | 0.999852266 | 1.003755362 | 0.070092219 | 0.001163891 |
| RAB1A | 1.001247691 | 0.997591766 | 1.004917015 | 0.504076499 | 0.051595534 |
| RPN1 | 0.998935093 | 0.997125339 | 1.000748132 | 0.249470909 | 0.069770389 |
| S100A10 | 1.001253023 | 1.000519474 | 1.001987111 | 0.000811592 | 0.016296974 |
| SAT1 | 1.000285056 | 0.99996455 | 1.000605664 | 0.081307197 | 0.007940965 |
| SEC61B | 1.001794007 | 0.999867739 | 1.003723986 | 0.067960475 | 0.015148216 |
| SH3YL1 | 0.998591692 | 0.983395808 | 1.014022389 | 0.857049797 | 0.160714175 |
| SNRPB2 | 1.012847556 | 1.000855377 | 1.024983426 | 0.035670816 | 0.013731681 |
| SPINT2 | 0.999462156 | 0.997592185 | 1.001335632 | 0.573401571 | 0.039776074 |
| SPOCK1 | 0.999006068 | 0.997992477 | 1.000020689 | 0.054855504 | 0.085504159 |
| SRP9 | 1.000441035 | 0.997876832 | 1.003011827 | 0.736305597 | 0.081534017 |
| SURF4 | 1.000132384 | 0.998217367 | 1.002051075 | 0.892318979 | 0.060570373 |
| TIMM17A | 1.020588814 | 1.00716903 | 1.034187405 | 0.002546735 | 4.92E-07 |
| TM9SF1 | 1.000499144 | 0.948891002 | 1.054914141 | 0.985265696 | 0.249306476 |
| TMEM63A | 0.996549623 | 0.987366639 | 1.005818014 | 0.464312312 | 0.14329652 |
| TNFSF10 | 0.998276505 | 0.996348553 | 1.000208187 | 0.080306384 | 0.001785753 |
| TOMM5 | 1.041901496 | 1.002221659 | 1.083152333 | 0.038266923 | 0.013729529 |
| UAP1 | 0.998539038 | 0.995989753 | 1.001094848 | 0.2622982 | 0.02406549 |
| YWHAQ | 0.999877535 | 0.998464443 | 1.001292627 | 0.865223317 | 0.050316866 |
| ALKBH7 | 1.000446038 | 0.998337343 | 1.002559186 | 0.678703517 | 0.033786504 |
| AZGP1 | 0.999809406 | 0.999612715 | 1.000006135 | 0.057583855 | 1.87E-05 |
| CBFA2T2 | 1.000817572 | 0.988639573 | 1.01314558 | 0.895906764 | 0.171971443 |
| CCDC85B | 1.004507813 | 1.000333121 | 1.008699928 | 0.034284293 | 0.000153674 |
| CD320 | 0.998087245 | 0.994421145 | 1.00176686 | 0.307852751 | 0.073412148 |

| | | | | | |
|---|---|---|---|---|---|
| CDK5RAP3 | 1.010578372 | 1.005607778 | 1.015573535 | 2.88E-05 | 3.53E-07 |
| CIRBP | 1.002144656 | 0.999653179 | 1.004642342 | 0.091634323 | 0.042809756 |
| DANCR | 1.000701884 | 0.997658915 | 1.003754134 | 0.651592297 | 0.069320908 |
| EEF2 | 0.999987581 | 0.999847264 | 1.000127917 | 0.862287962 | 0.135831577 |
| EIF3B | 1.002104644 | 0.99542614 | 1.008827955 | 0.537733467 | 0.048858325 |
| ERP29 | 0.998149401 | 0.996173474 | 1.000129248 | 0.066931737 | 0.009024712 |
| FAM189A2 | 0.992969176 | 0.984694135 | 1.001313757 | 0.098437503 | 0.000224443 |
| FAU | 1.000358973 | 0.999649085 | 1.001069366 | 0.321719043 | 0.018839833 |
| FLNB | 1.001870794 | 0.999493661 | 1.004253582 | 0.123050921 | 0.016233125 |
| HSD17B6 | 0.999970973 | 0.998794666 | 1.001148666 | 0.961448873 | 0.024205792 |
| IGFBP2 | 1.000516224 | 0.999118301 | 1.001916103 | 0.469400432 | 0.09679861 |
| KLK11 | 0.999993106 | 0.999198662 | 1.000788181 | 0.986435502 | 0.002694155 |
| KLK2 | 0.99998905 | 0.999837977 | 1.000140145 | 0.887039757 | 0.139023684 |
| MAP2K2 | 1.002888813 | 0.996913685 | 1.008899755 | 0.344083625 | 0.004697454 |
| MICOS13 | 1.003803356 | 0.999108937 | 1.008519832 | 0.112460591 | 0.005352127 |
| MZT2B | 1.000575783 | 0.998803854 | 1.002350855 | 0.524448281 | 0.01402703 |
| NAA10 | 1.010440323 | 0.997364845 | 1.02368722 | 0.118074941 | 0.000161585 |
| NACA | 1.000785762 | 0.998709204 | 1.002866637 | 0.458593536 | 0.195457297 |
| NDUFA11 | 1.000996153 | 0.992308133 | 1.009760241 | 0.822865614 | 0.06950174 |
| NDUFB11 | 1.001589738 | 0.99984624 | 1.003336276 | 0.073941303 | 0.00112841 |
| NISCH | 1.005838012 | 0.996366204 | 1.015399862 | 0.227878116 | 0.074103117 |
| NUMA1 | 1.002247712 | 0.998094595 | 1.006418112 | 0.289261435 | 0.034589836 |
| PABPC1L | 1.04694028 | 1.027389008 | 1.066863614 | 1.85E-06 | 3.88E-08 |
| PEBP1 | 1.000184498 | 0.999298257 | 1.001071524 | 0.683360188 | 0.02315852 |
| PILRB | 1.063416341 | 1.029171929 | 1.098800193 | 0.000231637 | 9.02E-06 |
| PIN1 | 1.009351489 | 0.999296846 | 1.019507298 | 0.068415943 | 0.002692829 |
| PLEKHH1 | 1.003059654 | 0.996355543 | 1.009808874 | 0.371928137 | 0.044950045 |
| POLD4 | 1.005143087 | 0.992052718 | 1.018406187 | 0.44308706 | 0.063342686 |
| RACK1 | 1.000142861 | 0.999537087 | 1.000749002 | 0.643998093 | 0.128268039 |
| S100A13 | 0.999346413 | 0.987239752 | 1.01160154 | 0.916269885 | 0.013591017 |
| SMS | 1.000050837 | 0.999809399 | 1.000292334 | 0.679863259 | 0.058315953 |
| SND1 | 0.998546963 | 0.995830352 | 1.001270985 | 0.295496259 | 0.024236742 |
| SNHG7 | 1.008440359 | 1.002557103 | 1.014358139 | 0.004871161 | 0.001257994 |
| SORD | 0.999738066 | 0.998309849 | 1.001168326 | 0.719480991 | 0.109690738 |
| TIMM13 | 1.001691993 | 0.998867471 | 1.004524502 | 0.240625002 | 7.95E-05 |
| TM7SF2 | 1.000697561 | 0.99834857 | 1.003052079 | 0.560867023 | 0.050726644 |
| VSTM2L | 1.000250783 | 0.999680803 | 1.000821088 | 0.388566863 | 0.048864737 |

HR: hazard ratio; HR.95L: low value of HR 95% confidence interval; HR.95H: high value of HR 95% confidence interval; P value: P value of Cox regression analysis; KM: P value of Kaplan-Meier analysis.

**Supplementary Table 7. Diverse trend towards in the expression of integrated genes from patient 1 in the advanced-stage parameters (tumor, GS > 7, pT3, TP53 mutation, prognosis outcomes of HR > 1 in RFS and PFS) and GS progression in the ST analysis. Related to Figure 3, 4.**

| Gene | Tumor | GS > 7 | pT3 | RFS HR > 1 | PFS HR > 1 | TP53 Mutation | GS upgrading (ST) |
|---|---|---|---|---|---|---|---|
| CNIH4 | Down | Up | Up | Up | Up | Up | Down |
| CRIP2 | Down | Up | Up | Up | Up | Up | Down |
| NANS | Up | Down | Down | Down | Down | Down | Down |
| PABPC1L | Up | Up | Up | Up | Up | NS | Up |
| PILRB | Up | Up | Up | Up | Up | Up | Up |
| DEGS1 | Up | Up | Up | NS | Up | Up | Down |
| ERP29 | Up | Down | Down | Down | NS | Down | Up |
| F5 | Up | Up | Up | Up | NS | NS | Down |
| FAM189A2 | Up | Down | Down | Down | NS | NS | Up |
| ISG15 | Up | NS | Up | Up | Up | NS | Down |
| MT1X | Down | Down | Down | Down | NS | Down | Down |
| SAT1 | Up | Up | Up | Up | NS | Up | Down |
| SNHG7 | Up | Up | Up | NS | Up | NS | Up |
| TIMM17A | Up | Up | Up | NS | Up | NS | Down |
| TOMM5 | Up | Up | Up | NS | Up | NS | Down |
| ANXA5 | Up | Up | Up | NS | NS | NS | Down |
| ARPC1B | NS | Up | Up | Up | NS | NS | Down |
| AZGP1 | NS | Down | Down | Down | NS | Down | Up |
| CACYBP | Down | Up | Up | NS | NS | NS | Down |
| CCDC85B | Up | NS | Up | NS | Up | NS | Up |
| CD320 | Up | Down | Down | NS | NS | Down | Up |
| CD46 | Down | NS | Down | NS | Down | NS | Down |
| CDK5RAP3 | Up | NS | NS | Up | Up | NS | Up |
| FKBP5 | NS | Down | Down | NS | Down | NS | Down |
| GPR160 | Up | Up | Up | NS | NS | Up | Down |
| HNRNPC | Up | Up | Up | NS | NS | Up | Down |
| HSD17B6 | NS | Down | Down | Down | NS | Down | Up |
| IGFBP2 | Up | Down | Down | NS | NS | Down | Up |
| KLK2 | Up | Down | Down | NS | NS | Down | Up |
| MORF4L1 | Down | Up | Up | NS | NS | Up | Down |
| MT1E | NS | Down | Down | Down | NS | Down | Down |
| SNRPB2 | NS | Up | Up | NS | Up | Up | Down |

Up: upregulation in the advanced stages or HR > 1 in survival analysis; Down: downregulation in the

advanced stages or HR > 1 in survival analysis; NS: no significance; ST: spatial transcriptomics.

**Supplementary Table 8. The expression levels of GS development related genes from patient 2 in TCGA-PRAD and normal tissues. Related to Figure 6.**

| Gene | Normal (median) | Tumor (median) | pValue | P symbol[a] |
|---|---|---|---|---|
| ASAH1 | 7.465753583 | 7.364302777 | 0.089008799 | ns |
| MIR99AHG | 2.949436065 | 2.667122558 | 0.003282313 | ** |
| PART1 | 4.019661581 | 4.623610552 | 0.000348726 | *** |
| PPFIA2 | 0.828478142 | 0.753258967 | 0.864815084 | ns |
| SESN3 | 4.49946704 | 4.31378132 | 0.384048153 | ns |

[a] ns P > 0.05; ** P < 0.01; ***P < 0.001.

**Supplementary Table 9. Expression levels of GS development related genes from patient 2 in TCGA-PRAD data stratified by GS. Related to Figure 6.**

| Gene | GS < 7 (median) | GS = 7 (median) | GS > 7 (median) | P (GS < 7 vs. GS > 7) | P symbol[a] |
|---|---|---|---|---|---|
| ASAH1 | 155.1954 | 170.99065 | 155.68095 | 0.561369498 | ns |
| MIR99AHG | 5.317893 | 5.0064665 | 5.8897645 | 0.146495668 | ns |
| PART1 | 28.80049 | 26.052355 | 19.003095 | 0.000124549 | *** |
| PPFIA2 | 0.3819781 | 0.5543748 | 1.242845 | 1.13161E-05 | *** |
| SESN3 | 13.19802 | 17.795425 | 22.90334 | 2.38931E-06 | *** |

[a] ns P > 0.05; ***P < 0.001.

**Supplementary Table 10. Expression levels of GS development related genes from patient 2 in TCGA-PRAD data stratified by pT. Related to Figure 6.**

| Gene | pT = 2 (median) | pT = 3 (median) | pT = 4 (median) | P (pT = 2 vs. pT = 3) | P symbol[a] |
|---|---|---|---|---|---|

| | | | | | |
|---|---|---|---|---|---|
| ASAH1 | 177.8803 | 157.1431 | 135.18945 | 0.009976647 | ** |
| MIR99AHG | 5.40996 | 5.259197 | 4.868744 | 0.737742649 | ns |
| PART1 | 28.2936 | 20.29418 | 17.87563 | 4.95388E-07 | *** |
| PPFIA2 | 0.4520994 | 1.036379 | 5.179435 | 3.57096E-07 | *** |
| SESN3 | 15.70823 | 21.48641 | 46.760595 | 4.23968E-06 | *** |

[a] ns P > 0.05; ** P < 0.01; ***P < 0.001.

**Supplementary Table 11. Expression levels of GS development related genes from patient 2 in TCGA-PRAD data stratified by TP53 mutation. Related to Figure 6.**

| Gene | Wild type (median) | Mutation (median) | P (wild vs. mut) | P symbol[a] |
|---|---|---|---|---|
| ASAH1 | 1.64E+02 | 150.0145 | 0.641532889 | ns |
| MIR99AHG | 5.259197 | 5.31069 | 0.692052968 | ns |
| PART1 | 23.99708 | 19.787525 | 0.029119687 | * |
| PPFIA2 | 0.652031 | 1.3321575 | 0.018094224 | * |
| SESN3 | 18.24143 | 24.26451 | 0.011581015 | * |

[a] ns P > 0.05; * P < 0.05.

**Supplementary Table 12. Survival analysis and univariate Cox regression analysis of GS development related genes from patient 2 in RFS outcomes. Related to Figure 6.**

| Gene | HR | HR.95L | HR.95H | P value | KM |
|---|---|---|---|---|---|
| ASAH1 | 0.998868557 | 0.995863539 | 1.001882643 | 0.461466702 | 0.069401481 |
| MIR99AHG | 1.019045875 | 0.972151781 | 1.06820202 | 0.432493862 | 0.029690647 |
| PART1 | 0.989261891 | 0.974310875 | 1.004442335 | 0.164683216 | 0.008644551 |
| PPFIA2 | 1.016857348 | 1.001810945 | 1.032129736 | 0.027960358 | 0.000412125 |
| SESN3 | 1.002308734 | 0.996323575 | 1.008329846 | 0.450457496 | 0.051958619 |

HR: hazard ratio; HR.95L: low value of HR 95% confidence interval; HR.95H: high value of HR 95% confidence interval; P value: P value of Cox regression analysis; KM: P value of Kaplan-Meier analysis.

**Supplementary Table 13. Survival analysis and univariate Cox regression analysis of GS development related genes from patient 2 in PFS outcomes. Related to Figure 6.**

| Gene | HR | HR.95L | HR.95H | P value | KM |
|---|---|---|---|---|---|
| ASAH1 | 0.998266138 | 0.995778284 | 1.000760207 | 0.172859371 | 0.007161131 |
| MIR99AHG | 1.027934032 | 0.991560503 | 1.065641856 | 0.133905866 | 0.008084487 |
| PART1 | 0.986807855 | 0.974593511 | 0.999175279 | 0.036635778 | 5.06E-05 |
| PPFIA2 | 1.020836051 | 1.009472054 | 1.032327977 | 0.000305541 | 3.36E-07 |
| SESN3 | 1.005827942 | 1.001595533 | 1.010078235 | 0.006913522 | 0.010361351 |

HR: hazard ratio; HR.95L: low value of HR 95% confidence interval; HR.95H: high value of HR 95% confidence interval; P value: P value of Cox regression analysis; KM: P value of Kaplan-Meier analysis.

**Supplementary Table 14. Diverse trend towards in the expression of integrated genes from patient 2 in the advanced-stage parameters (tumor, GS > 7, pT3, TP53 mutation, prognosis outcomes of HR > 1 in RFS and PFS) and GS progression in the ST analysis. Related to Figure 6.**

| Gene | Tumor | GS > 7 | pT3 | RFS HR > 1 | PFS HR > 1 | TP53 Mutation | GS upgrading (ST) |
|---|---|---|---|---|---|---|---|
| PART1 | Up | Down | Down | NS | Down | Down | Up |
| PPFIA2 | NS | Up | Up | Up | Up | Up | Up |
| SESN3 | NS | Up | Up | NS | Up | Up | Up |
| ASAH1 | NS | NS | Down | NS | NS | NS | Up |
| MIR99AHG | Down | NS | NS | NS | NS | NS | Up |

Up: upregulation in the advanced stages or HR > 1 in survival analysis; Down: downregulation in the advanced stages or HR > 1 in survival analysis; NS: no significance; ST: spatial transcriptomics.
